# The influence of tobacco use, hazardous drinking, and other risk factors on HPV-associated oropharyngeal cancer risk and screening perceptions among gay and bisexual men: a cross-sectional study

**DOI:** 10.1186/s12903-025-05774-0

**Published:** 2025-03-30

**Authors:** I. Niles Zoschke, Sarah L. Bennis, Yi Tang, J. Michael Wilkerson, Cynthia L. Stull, Alan G. Nyitray, Samir S. Khariwala, C. Mark Nichols, B. R. Simon Rosser, Charlene A. Flash, Michael W. Ross

**Affiliations:** 1 Alcohol Research Group, University of California at Berkeley, Berkeley, CA US; 2 Division of Epidemiology and Community Health, School of Public Health, University of Minnesota, Minneapolis, MN USA; 3 Department of Biostatistics and Data Science, School of Public Health, The University of Texas Health Science Center at Houston, Houston, TX USA; 4 Department of Health Promotion and Behavioral Science, School of Public Health, The University of Texas Health Science Center at Houston, Houston, TX USA; 5 Department of Primary Dental Care, School of Dentistry, Division of Dental Hygiene, University of Minnesota, Minneapolis, MN USA; 6 Cancer Center, Medical College of Wisconsin, Milwaukee, WI USA; 7 Center for AIDS Intervention Research, Medical College of Wisconsin, Milwaukee, WI USA; 8 Department of Otolaryngology-Head and Neck Surgery, University of Minnesota, Minneapolis, MN USA; 9 Avenue 360 Health and Wellness, Houston, TX USA; 10 Department of Family Medicine, School of Medicine, University of Minnesota, Minneapolis, MN USA

**Keywords:** LGBTQ, Gay, Cigarette smoking, Vaping, Alcohol use, Oral sex, Oral health, Oropharyngeal cancer, Health education, Men who have sex with men, HPV

## Abstract

**Background:**

Oropharyngeal cancer is the eighth most common cancer among US men and its incidence is sharply rising. Oropharyngeal cancer manifests in two major ways: the classic form is prevalent among people who use alcohol and tobacco heavily, while a growing subset of incident cases is associated with human papillomavirus-16 (HPV) and transmitted via oral sex. Gay and bisexual men appear at higher risk for each etiologic subset of oropharyngeal cancer than heterosexual men. We conducted a cross-sectional study to learn how tobacco use, hazardous drinking, and other key risk factors affect gay and bisexual men’s perceptions of oropharyngeal cancer risk and beliefs about screening at a doctor’s office and self-screening at home.

**Methods:**

We recruited 1,699 gay and bisexual men from two dating websites to participate in an online survey. We asked about tobacco use, alcohol consumption, sexual history, and other risk factors for oropharyngeal cancer. The survey also investigated participants’ perceptions of oropharyngeal cancer risk and potential worry related to screening. We analyzed results at the bivariate level and in multivariable regression models. We used logistic regression to analyze categorical data and linear regression to analyze continuous data.

**Results:**

Average age of participants was 41.5 (SD = 12.7) years. Most were cisgender (95%), and identified as gay (80%), while 19% were bisexual or pansexual, with 2% reporting being queer or a self-described sexuality. Factors associated with high perceived oropharyngeal cancer risk perceptions were cigarette smoking, using both cigarettes and vaping, being gay identified, number of sexual partners in the last 12 months, and having poor mouth/teeth condition. Factors associated with oropharyngeal cancer screening worry were being Hispanic, having queer/self-described sexuality, not having health insurance, and having poor mouth/teeth condition. No factors were associated with self-screening at home. Alcohol use was not associated with oropharyngeal cancer risk perception.

**Conclusions:**

This study examines oropharyngeal cancer risk perceptions among gay and bisexual men. Health promotion efforts to reduce oropharyngeal cancer risk among gay and bisexual men should involve comprehensive oral health, sexual health, and tobacco use education. Researchers should continue investigating acceptable and effective home self-screening methods for HPV-associated cancers.

**Supplementary Information:**

The online version contains supplementary material available at 10.1186/s12903-025-05774-0.

## Introduction

Oropharyngeal cancer (OPC) affects the soft palate, the rear of the tongue, the oropharynx walls, the uvula, and the tonsils. Oropharyngeal cancer has two etiologic subtypes: the classic type is prevalent among people who drink alcohol and use tobacco heavily, while the other type is predominantly associated with human papillomavirus (HPV)-16 and is associated with oral sex [1, 2]. The incidence of OPC is sharply rising, making it the eighth most common cancer among US men [3]. Gay and bisexual men (GBM) have a higher prevalence of alcohol use and tobacco use, relative to heterosexual individuals [4] and relative to the general population [5]. Men who report sexual contact with other men also have a higher prevalence of oral HPV infections relative to those who don’t [6–7]. While HPV is the main driver of OPC among men [8], the combination of high rates of alcohol use, tobacco use, and HPV infections may put GBM at higher risk for an OPC.

Human papillomavirus-associated OPC is much more survivable than classic OPC [9]. A recent US study found that overall survival rates at two years for HPV-associated OPC cases compared to classic cases were 93.1 vs. 77.8% (*p* < 0.001) [10]. Finding and treating HPV-associated invasive OPC before cancer metastasis reduces morbidity and mortality substantially [11]. Currently, the OPC precancer cannot be identified relegating treatment for individuals only after invasion. Moreover, there is no recommended screening for oropharyngeal precancers as there is for HPV-associated cancers of the cervix [12]. Finding and treating OPC early may reduce distress and improve the quality of life for people diagnosed with cancer [11]. Given the sharp increase in OPC increase and the looming threat it poses to GBM health equity, it is necessary to understand how GBM perceive their risk for OPC how they view screening approaches, especially among those who drink alcohol and use tobacco heavily.

Heavy alcohol use among GBM varies by race/ethnicity, numbers of sexual partners, education level [13] health as a value, and age [14]. Tobacco use among gay and bisexual men varies by race/ethnicity, alcohol consumption [15] healthcare coverage [16], age, and education [17]. Beyond alcohol, tobacco use, and HPV infections, risk factors for head and neck cancers also include poor dentition, infrequent tooth brushing, and infrequent dental visits [18, 19] It is well-established that tobacco use in any form is associated with several oral health conditions, including periodontal disease and tooth loss [20]. Moreover, people who smoke greater numbers of tobacco products and for longer periods are at increased risk for periodontal disease [20].

Very little is known about how levels of tobacco use, alcohol use, and oral health influences how GBM assess their own cancer risk. Investigating this topic is especially important considering recent research demonstrating alarmingly high rates of e-cigarette use among lesbian, gay, bisexual, and transgender people [21]. Accurate self-assessment for risk and accurate health information may motivate GBM to seek screening and early detection for OPC. Our analysis aims to shed light on the factors that influence how GBM perceive their own OPC risk and potential screening approaches for OPC. In response to the sharp increase in HPV-associated OPC incidence, particularly among GBM [6, 22, 23], we analyzed data from an online sample of 1,699 GBM. We collected data on both classic OPC and HPV-associated OPC risk factors and risk perceptions. This analysis reports on how various risk factors such as tobacco use, and hazardous drinking may influence how GBM perceive their risk for OPC.

## Methods

This analysis was conducted using cross-sectional data from an online sample of GBM. All study materials and methods were approved by the University of Minnesota Institutional Review Board. We recruited GBM from two online dating apps, Scruff and Jack’d (Perry Street Software Inc., New York, NY). We aimed to recruit 1700 respondents, to allow for an estimated 33% of participants to not fully complete the survey. Between February and March 2022, we ran study advertisements on Scruff and Jack’d. Every user based in the geotargeted area who logged into either app during this period was shown the study advertisement, which could be saved as a message on the app as well. The campaign ran until we recruited our target sample size. When a respondent clicked on the link, they were connected to the study pre-screening survey to determine eligibility.

All study participants had to be GBM older than 18 years who lived in the US. They must also have had sex with a man in the previous five years. Transgender men, nonbinary people, and other self-identifying gender categories who also identified as GBM were eligible to participate. If respondents met the study inclusion criteria and chose to participate in the study, they were connected to a consent form. Of 4,464 people who initiated the consent form, 1,836 respondents completed it, a total of 19.86% of the unique advertisement clicks.

After the recruitment period, we reviewed survey responses to ensure they represented unique people and not online bots, adapting an established cross-validation and deduplication protocol for our study [24]. We removed 114 responses during the cross-validation and deduplication process, leaving 1,722 valid and unique responses and 1,699 participants who completed at least the first survey question. Valid participants who completed the survey were offered $50 compensation.

### Measures

#### Tobacco use

The questionnaire applied in this study was developed purposely for this study and can be found in the supplementary material. We adapted our tobacco use items and variables from the Population Assessment of Tobacco and Health (PATH) study [25]. Similar tobacco use measurement has been applied in several recent studies among sexual and gender minority populations [26–28]. The PATH instrument assesses lifetime prevalence of a number of tobacco use categories, if the participant meets a threshold of lifetime use to be considered a user of that product, and last 30-day incidence of use [25].

To determine cigarette use, we asked the yes or no question, “Have you smoked 100 cigarettes (5 packs) or more in your entire life?” If the response was yes, we asked “Have you smoked a cigarette in the past 30 days?” Participants who responded yes to this question and who did not also meet the criteria for other tobacco product use were categorized as “smokes cigarettes only.” To determine electronic nicotine product use, we asked the yes or no question, “Have you used electronic nicotine products 100 or more times in your entire life?” If the response was yes, we asked “Have you used an electronic nicotine product in the past 30 days?” with a yes or no response. Participants who responded yes to this question and who did not also meet the criteria for other tobacco product use were categorized as “uses electronic nicotine products only”. The category “dual use” was assigned to any participant who met the use criteria for both smoking cigarettes and using electronic nicotine products. Participants who were determined to be hookah users (*N* = 9), pipe smokers (*N* = 2), snus users (*N* = 1), and smokeless tobacco users (*N* = 11) were dropped from the analysis and not included in the “dual use” category due to low numbers of respondents. All tobacco use categories are mutually exclusive. We applied the tobacco use category “not a tobacco user” for people who did not meet the lifetime product consumption threshold for any category or if participants indicated they had not used a tobacco product in the past 30 days.

#### Hazardous drinking

We applied The Alcohol Use Disorder Identification Test (AUDIT-C) to determine hazardous drinking among study participants [29, 30]. The AUDIT-C was developed with the World Health Organization and applies a three-item scale to examine past 12-month alcohol use behaviors, including drinking frequency, binge drinking frequency, and the amount of alcohol consumed per day [29]. People assigned male at birth scoring four or above and people assigned female at birth scoring three or above were considered hazardous drinkers (nationally representative US sample areas under receiver operator characteristic curves [AUROCs] = 0.86 for any alcohol use disorder, 0.89 for alcohol dependence, and 0.97 for risk drinking) [31].

#### Sociodemographic characteristics

We measured age, race and ethnicity, sex assigned at birth, current gender, sexuality, education, and annual income. We considered age as a continuous variable and all other demographic variables categorical. We conceptualized race/ethnicity using the categories White non-Hispanic, African American / Black non-Hispanic, Asian non-Hispanic, Hispanic, and Other. We collected data for multiple current gender categories (i.e., cisgender man, cisgender woman, transgender man, transgender woman, non-binary/gender non-conforming and also included an option to self-describe. Cisgender women were not included in analysis. We collapsed transgender men, trans-masculine, non-binary, and two-spirit/hijra responses into one category due to low numbers of respondents. The resulting categories were “cisgender men” and one category titled “Trans man, non-binary, or self-described” encompassing all participants who responded as transgender men, non-binary (includes trans-masculine non-binary, and two-spirit/hijra) and self-described with an open response format. Sexual orientation categories at data collection were “gay,” “bisexual,” “pansexual/panamorous,” and self-described with an open response format. We collapsed the “bisexual” and “pansexual” categories into a new category titled “bisexual or pansexual” due to low sample size of pansexual responses. We also collapsed the self-described categories due to the low sample size among some of the original responses. We collected education with the following categories: “less than high school,” “high school graduate or GED,” “some college but no degree,” “associate’s degree,” “bachelor’s degree,” and “graduate or professional degree.” We recategorized these responses as “less than high school or GED, and less than high school,” “some college,” and “college degree or higher” due to low sample size in some of the categories.

#### Health risk characteristics

We constructed the “last 12 months of sexual partners” variable as a continuous variable measuring the number of cisgender men a participant had sex with in the past 12 months. We treated health insurance status as a dichotomous variable. We determined the “self-described mouth and teeth condition” variable by asking the question, “How would you describe the condition of your mouth and teeth?” The response options included “poor,” “fair,” “good,” and “very good” which were dichotomized into “poor,” including the “poor” and “fair” categories and “good” including the “good” and “very good” categories.

#### OPC risk and screening perceptions

The primary dependent variable for this analysis was risk perception for OPC. We operationalized this into three participant risk perceptions: subjective risk of OPC, whether participants would be worried about what a doctor may find if screened for OPC, and if participants would feel comfortable doing an OPC self-examination at home. Our research team developed these items for the present research applying concepts from the Health Belief Model [32], which includes perceptions of risk, and positive and negative feelings, in its calculation [33]. The questions were phrased as, “I believe I have several risk factors for oropharyngeal cancer,” “I’m scared of what a healthcare provider (like a doctor or a dentist) might find if I get screened for oropharyngeal cancer,” and “I would feel comfortable getting checked for oropharyngeal cancer if I could do a self-screening at home.” These items were scored using a 5-point Likert scale ranging from strongly disagree to strongly agree. We dichotomized responses to each item into high (somewhat to strongly agree) and low (strongly disagree to neither agree nor disagree) categories in the case that Likert responses were skewed towards a small number of categories.

### Statistical analysis

We used STATA version 17 to conduct all analyses [34]. We examined the relationship between all independent variables for all three dependent variables in this model. We used chi-squared tests for most of the categorical bivariate analyses, except when analyses lacked sufficient power or contained empty cells, in which case we used Fisher’s exact tests to assess differences. Some participants did not respond to all the questions involved in various tests and therefore were missing data. Data missingness influenced some of the variables used in this analysis. We attempted to use statistical approaches to impute missing data; however, these approaches did not benefit analytical modeling. Therefore, for each participant, we dropped from analysis only the missing values of variables used in each model, but used all other data that was not missing for each model. For example, if a participant did not provide a response for age, but they did provide a response for race, we used the race data and not the age data for each relevant model. The variable representing the number of sexual partners participants had outliers, so we opted to log transform this variable for analysis. Because some participants reported zero sexual partners, which would result in an undefined value, we took the log of sexual partners plus a very small number (0.00001). This ensured that the shape of the covariate curve stayed the same while being defined for all observations.

We used *p* < 0.10 (2-tailed) to identify candidate variables for inclusion in the bivariate analyses. All factors which were found significant for at least one of the outcomes, as well as the main predictor variables tobacco use, and hazardous drinking, were applied in a logistic regression model controlling for other factors found significant at the bivariate level. Because this was an exploratory study, we did not analyze results using a focal variable. We did not adjust results.

## Results

A total of 1,699 participants met eligibility criteria, completed at least the first question, and were determined to be valid responses and not online “bot” responses through our cross-validation and response de-duplication process. Bivariate demographic data for each outcome of interest are summarized in Table 1. Regression analyses are summarized in Table 2. Note that for the last column in the tables we reverse-scored the results and thus reported results for the question “I would feel comfortable getting checked for oropharyngeal cancer if I could do a self-screening at home” as “I would feel uncomfortable getting checked for oropharyngeal cancer if I could do a self-screening at home” so that the effect sizes of all the columns could all be interpreted in the same direction.

**Table 1:** Participant characteristics (*N* = 1699)

Demographic characteristics	Overall	High OPC risk perceptions			Worried about what a doctor might find if screened for OPC			Uncomfortable getting checked for OPC doing a self-screening at home		
	1699	No *n* = 941 (55.4%)	Yes *n* = 566 (33.3%)		No *n* = 1110 (65.3%)	Yes *n* = 381 (22.4%)		No *n* = 1364 (80.3%)	Yes *n* = 125 (7.4%)	
	Mean (sd)	Mean (sd)	Mean (sd)	p value	Mean (sd)	Mean (sd)	p value	Mean (sd)	Mean (sd)	p value
**Age**	41.48 (12.67)	41.57 (12.74)	42.15 (12.34)	0.390	42.44 (12.64)	39.99 (12.27)	**0.00**	42.05 (12.59)	39.56 (12.28)	**0.03**
Past 12 months sexual partners*	1.61 (2.56)	1.46 (2.61)	1.87 (2.43)	**0.00**	1.59 (2.57)	1.67 (2.47)	0.78	1.77 (2.41)	1.60 (2.56)	0.47
	n (%)	n (%)	n (%)	p value	n (%)	n (%)	p value	n (%)	n (%)	p value
**Race/Ethnicity**				**0.00**			**0.00**			0.28
White non-Hispanic	969 (58.7)	561 (61.1)	332 (59.3)		696 (64.0)	190 (51.0)		821 (61.4)	64 (52.9)	
African American / Black non-Hispanic	277 (16.8)	145 (15.8)	87 (15.6)		160 (14.7)	64 (17.2)		200 (15.0)	24 (19.8)	
Asian non-Hispanic	53 (3.2)	25 (2.7)	23 (4.1)		32 (2.9)	16 (4.3)		45 (3.4)	3 (2.5)	
Hispanic	252 (15.3)	141 (15.4)	76 (13.6)		133 (12.2)	82 (22.0)		191 (14.3)	23 (19.0)	
Other**	100 (6.1)	46 (5.0)	41 (7.3)		66 (6.1)	21 (5.6)		80 (6.0)	7 (5.8)	
**Gender assigned at birth**				0.44			1.00			0.64
Male	1679 (99.0)	929 (98.7)	560 (99.3)		1096 (98.9)	377 (99.0)		1348 (99.0)	123 (98.4)	
Female	17 (1.0)	12 (1.3)	4 (0.7)		12 (1.1)	4 (1.0)		14 (1.0)	2 (1.6)	
**Current gender**				0.23			0.50			0.82
Cisgender man	1604 (95.2)	889 (94.6)	541 (96.1)		1058 (95.5)	358 (94.5)		1296 (95.3)	118 (94.4)	
Trans man, non-binary, or self-described***	80 (4.8)	51 (5.4)	22 (3.9)		50 (4.5)	21 (5.5)		64 (4.7)	7 (5.6)	
**Sexuality**				0.21			**0.01**			0.45
Gay	1349 (79.8)	751 (79.9)	473 (83.6)		914 (82.4)	298 (78.2)		1102 (80.9)	107 (85.6)	
Bisexual or pansexual	313 (18.5)	173 (18.4)	85 (15.0)		183 (16.5)	71 (18.6)		239 (17.5)	16 (12.8)	
Queer or other self-described**†**	28 (1.7)	16 (1.7)	8 (1.4)		12 (1.1)	12 (3.2)		22 (1.6)	2 (1.6)	
**Education**				0.24			0.12			0.15
High school or GED, and less than high school	147 (8.7)	83 (8.8)	39 (6.9)		82 (7.4)	38 (10.0)		104 (7.6)	13 (10.5)	
Some college	399 (23.7)	218 (23.2)	121 (21.4)		241 (21.7)	92 (24.2)		299 (22.0)	34 (27.4)	
College degree or higher	1140 (67.6)	639 (68.0)	406 (71.7)		786 (70.9)	250 (65.8)		960 (70.4)	77 (62.1)	
**Do you currently have health insurance?**				0.77			**0.00**			0.28
No	109 (8.2)	71 (8.7)	38 (0.08)		61 (6.2)	48 (14.2)		96 (7.9)	13 (11.3)	
Yes	1216 (91.8)	747 (91.3)	464 (0.92)		927 (93.8)	289 (85.8)		1114 (92.1)	102 (88.7)	
**How would you describe the condition of your mouth and teeth?**				**0.03**			**0.00**			0.13
Good	1157 (72.7)	721 (76.7)	381 (0.67)		851 (76.7)	242 (63.7)		1007 (73.9)	84 (67.2)	
Poor	435 (27.3)	219 (3.3)	185 (0.33)		258 (23.3)	138 (36.3)		355 (26.1)	41 (32.8)	
**Tobacco use (past 30 days)**				**0.00**			0.21			0.70
Not a tobacco user	1085(78.3)	576 (80.1)	297 (0.66)		644 (76.0)	211 (69.0)		776 (73.6)	76 (77.6)	
Smokes cigarettes only	194 (14.0)	88 (12.2)	104 (0.23)		126 (14.9)	67 (21.9)		181 (17.2)	13 (13.3)	
Vapes only	72 (5.2)	43 (6.0)	29 (0.06)		55 (6.4)	17 (5.5)		65 (6.2)	7 (7.1)	
Dual use (two or more of any category)	34 (2.5)	12 (1.7)	22 (0.05)		23 (2.7)	11 (3.6)		32 (3.0)	2 (2.0)	
**Alcohol use (Audit C)**				0.43			0.60			0.11
Not a hazardous drinker	1248 (88.4)	776 (89.0)	467 (0.87)		931 (88.8)	316 (87.5)		1136 (88.0)	112 (93.3)	
Hazardous drinker	163 (11.6)	96 (11.0)	67 (0.13)		118 (11.2)	45 (12.5)		155 (12.0)	8 (6.7)	

Note: Differences in counts are the result of missing data. Bolded values indicate statistical significance at *p* < 0.10

*Used the log of past 12 months sex partners

**Other responses included American Indian, Alaska Native, Native Hawaiian, Pacific Islander, other non-Hispanic, 2 or more races

***Self-described responses included “post-binary,” “gay,” “pansexual,” “omnisapien,” and “man without cisgender qualification.”

**†**Self-described responses included “queer,” “questioning,” “demisexual,” “no labels” and “other” and were combined into the category

**Table 2:** Multivariable regression model of differences in association OPC risk perception among types of tobacco and alcohol users

Regression models	High OPC risk perceptions *n* = 941 (55.4%)	Worried about what a doctor might find if screened for OPC *n* = 1110 (65.3%)	Uncomfortable getting checked for OPC doing a self-screening at home *n* = 1364 (80.3%)
	aOR [95% CI]	aOR [95% CI]	aOR [95% CI]
**Tobacco**			
Not a Tobacco user	[Ref]	[Ref]	[Ref]
Cigarettes	**2.16 [1.49**,** 3.13]**	1.32 [0.88, 1.96]	1.25 [0.67, 2.51]
Vapes	1.21 [0.69, 2.07]	0.85 [0.44, 1.57]	0.87 [0.40, 2.22]
Dual Use	**4.19 [1.89**,** 10.02]**	1.11 [0.47, 2.49]	1.84 [0.51, 11.94]
**Alcohol use**			
Not hazardous drinking	[Ref]	[Ref]	[Ref]
Hazardous drinking	1.10 [0.74, 1.62]	0.85 [0.54, 1.31]	1.88 [0.90, 4.60]
**Race/Ethnicity**			
White, Non-Hispanic	[Ref]	[Ref]	[Ref]
African American	0.92 [0.60, 1.39]	1.15 [0.73, 1.78]	0.78 [0.41, 1.55]
Asian	1.00 [0.40, 2.37]	1.74 [0.68, 4.14]	1.05 [0.29, 6.77]
Hispanic	0.86 [0.58, 1.26]	**2.00 [1.34**,** 2.96]**	0.74 [0.41, 1.40]
Other	1.87 [1.03, 3.42]	0.82 [0.39, 1.60]	0.67 [0.28, 1.85]
**Age**	1.01 [1.00, 1.02]	0.99 [0.98, 1.01]	1.01 [0.99, 1.03]
**Sexual Orientation**			
Gay	[Ref]	[Ref]	[Ref]
Bisexual	**0.68 [0.46**,** 0.99]**	1.14 [0.76, 1.70]	1.35 [0.72, 2.77]
Queer/Self-described	1.08 [0.35, 3.09]	**3.16 [1.08**,** 8.96]**	1.55 [0.29, 28.55]
*Sexual Partners	**1.09 [1.03**,** 1.17]**	1.04 [0.98, 1.11]	0.96 [0.0.86,1.05]
**Health Insurance**			
No	[Ref]	[Ref]	[Ref]
Yes	1.61 [0.97, 2.73]	**0.50 [0.31**,** 0.82]**	1.53 [0.71, 3.03]
**Mouth/teeth condition**			
Good	[Ref]	[Ref]	[Ref]
Poor	**1.47 [1.08**,** 2.01]**	**1.65 [1.19**,** 2.29]**	0.70 [0.43, 1.15]

*Used the log of past 12 months sex partners

### Sociodemographic characteristics

Sociodemographic characteristics are displayed in Table 1. The average age of participants was 41.5 years old (SD = 12.7) Most of the participants were assigned male at birth (99%) and cisgender (95%), with 5% being transgender men, nonbinary, or self-described. Most were gay (80%), 19% were bisexual or pansexual, with 2% reporting being queer or a self-described sexuality. Just over half (59%) were White non-Hispanic, 17% Black non-Hispanic, and 15% were Hispanic with a small proportion of participants being Asian non-Hispanic or something else. Most participants (68%) had a college degree or higher.

### Health risk characteristics

The median number of sexual partners in the past 12 months was 7 (IQR: 3–20) Almost all (92%) participants reported having health insurance. Just over one out of four participants (27%) reported having poor mouth and teeth condition. Most study participants didn’t use tobacco (78%); 14% smoked cigarettes, 5% vaped only, and 2% smoked cigarettes and vaped. No participants met the threshold criteria for smoking cigars, filtered cigars, or cigarillos. Only 12% were categorized as hazardous drinkers.

### Bivariate associations of OPC risk and screening perceptions

In bivariate analysis, race/ethnicity, past 12 months of sexual partners, mouth/teeth condition, and tobacco use were associated with high OPC risk perceptions. Age, race/ethnicity, sexuality, health insurance status, and mouth/teeth condition were significantly associated with being worried about what a doctor may find if screened for OPC. Age was the only factor significantly associated with feeling uncomfortable getting checked for OPC at home.

### Multivariable regression model

Results from the multivariable regression model are displayed in Table 2. Factors associated with high OPC risk perceptions were cigarette smoking (aOR = 2.16 [1.49, 3.13]), dual tobacco use (aOR = 4.19 [1.89, 10.02]) having poor mouth/teeth condition (aOR = 1.47 [1.08, 2.01]) and number of sexual partners in the last 12 months (aOR = 1.09[1.03–1.17]). Because the reported number of sexual partners in the last 12 months was analyzed using log transformed data due to high skew, this result should be interpreted as when the log of sexual partners increases by 1, the odds of high OPC risk perceptions will increase by a factor of 1.09. Being bisexual was associated with reduced high OPC risk perceptions (aOR = 0.68 [0.46, 0.99]). Factors associated with being worried about what a doctor may find if screened for OPC were being Hispanic (aOR = 2.00 [1.34, 2.96]), having queer/self-described sexuality (aOR = 3.16 [1.08, 8.96]) and having poor mouth/teeth condition (aOR **=** 1.65 [1.19, 2.29]). Participants with health insurance had half the odds of reporting being worried about what a doctor may find if screened for OPC (aOR = 0.50 [0.31, 0.82]). No factors were associated with feeling uncomfortable self-screening for OPC at home.

## Discussion

This study surveyed GBM on OPC risk perceptions. Our purpose was to provide data that can be applied to health education for a critical population experiencing HPV-associated OPC disparities. A key finding of this analysis is that cigarette smokers had 2.16 higher odds of considering themselves at high risk for OPC compared to non-tobacco users. Participants who smoked cigarettes and vaped had 4.19 higher odds of considering themselves at high risk for OPC compared to non-tobacco users. Since our analyses demonstrate that both cigarette smoking and dual using GBM had higher odds of considering themselves at higher risk for OPC, this may indicate that health messaging on smoking and dual-use and their OPC risks are reaching GBM, a key strength to capitalize on in future health promotion efforts.

On the sexual factors, bisexual identified men had much lower odds than gay identified men to perceive themselves at high risk, and there was only a weak association between number of sexual partners, last 12 months, and perceived risk for OPC. Persistent oral HPV infection increases risk for OPC [35]. Several studies [22, 36] have reported that greater numbers of sexual partners and oral sex partners are associated with OPC. The strong self-perceptions of risk by smokers and dual use participants stand in contrast with the weak self-perceptions of risk among those more sexually active. This likely reflects the contrast in education between smoking and sexual partners as a risk factor for cancer. Participants appear well aware that smoking causes cancer (and possibly OPC), but less aware of sexual risk (at least as measured by sexual orientation and partner number). To address the rising incidence of OPC in GBM, education on sexual risk is needed.

Relatedly, our analysis demonstrated that GBM with poor self-rated mouth/teeth condition had 1.47 higher odds of perceiving they were at high risk for OPC, and 1.65 higher odds of being worried about what a doctor may find if checked for OPC compared to those with good self-rated mouth/teeth condition. Our study reports that GBM viewed the condition of their mouth/teeth as an important factor in OPC risk assessment, and this may be another viable opportunity for health education aimed at OPC early detection among this at-risk population. Based on previous work and our analysis, comprehensive oral health, sexual health, and tobacco use education appears to be a strategy worth exploring for preventing and detecting early OPC among GBM. Such education will likely have to overcome a structural barrier that neither dentists nor GBM patients may be used to discussing sexual health as part of oral health [37].

Participants had greater odds of reporting being worried about what a healthcare provider might find if they identified as queer/self-identified, were Hispanic, did not have health insurance, and if they described their mouth/teeth condition as poor. Participants who were queer/self-identified and Hispanic may be more likely to fear discrimination when seeking medical care while those without insurance face access concerns, discrimination, and ability to pay. Those with worse mouths may fear that cancer is already implicated in their poor mouth/teeth condition.

Bisexual participants had 1.32 times the odds of having high OPC risk perceptions, relative to gay men. While the results show that queer/self-described had 3.16 greater odds of being afraid of what a doctor may find if screened for OPC relative to gay men. This finding is likely an artifact of small cell counts and should be interpreted inconclusively as the confidence interval was large (aOR = 3.16 [1.08, 8.96]). In general, these findings raise important questions about why various groups of gay, bisexual, and queer/self-described men may vary in OPC risk perceptions and fears of screening. Perhaps outness or self-disclosure in healthcare settings varies among gay, bisexual, and queer/self-described categories among men, and perhaps this influences OPC risk perceptions and fears of screening. Ceres et al. [38] argue that gay or bisexual status disclosure is important as it allows clinicians to provide compassionate cancer care and proactive screening, a finding reinforced by Zoschke et al. [37] which found that oral healthcare providers reported GBM clients sometimes expressed shame or guilt surrounding sexual practices which impeded important OPC prevention activities. Future work should research the mechanisms that differentially affect gay, bisexual/pansexual, and queer/self-described men to account for differences in OPC risk and beliefs about screening. This may require researchers to use within-group recruitment stratification to ensure representation from different sexual identities.

Hispanic participants had twice the odds of reporting being worried about what a doctor may find if screened for OPC relative to White men. Recent research has demonstrated that Hispanic males living in the United States have higher rates of HPV-associated OPC-specific mortality even adjusted for treatment differences between race/ethnic categories [39]. This research called for increased early detection to reduce racial/ethnic disparities in OPC mortality and further research on factors that influence OPC outcomes [39].

Hazardous drinking influenced none of the outcome variables in the overall multivariable models. Being cautious not to infer a finding from a null result, this lack of association raises questions about risk and screening perceptions among hazardous drinking GBM considering alcohol use is a key risk factor for classic and HPV-associated OPC [13]. Future health promotion efforts should consider ways to tailor communications to highlight hazardous drinking as a risk factor for OPC to GBM to increase perceived susceptibility to OPC and reduce OPC disparities.

One important consideration of our analysis is that the average age of participants was 41.5 years old. In 2019, the Advisory Committee on the Immunization Practices changed the age recommendations for the HPV vaccines [40], based on findings that the vaccine is effective for people ages 27–45 who are at higher risk [41]. Recent research found that nearly two-thirds of Americans in this age bracket were not aware that HPV causes cancers other than cervical cancer [41]. Other recent research has showed while only 27.2% of a participants in a nationally representative US-based study were aware that HPV causes anal cancer, gay men had 2.27 the odds of being aware of the connection compared to heterosexual men [OR 2.27; 95% CI (1.24, 4.14); 42]. Bisexual men participating in the same study were not more aware than heterosexual men, suggesting that awareness of the link between HPV and anal cancer is not uniform between gay and bisexual men. Based on the recruitment success of our study, future research might be able to recruit a GBM using the same methods to reach HPV vaccination-eligible GBM to promote this cancer prevention strategy paying special attention to differences in the education needs and preference of both gay and bisexual men.

Lastly, our analyses found that factors influencing OPC risk were not associated with participants’ comfort with getting checked for OPC at home. These results demonstrate that the study population across the board felt comfortable with OCP self-screening, regardless of tobacco use status, hazardous drinking, race/ethnicity, age, sexual orientation, past 12 months of sexual partners, health insurance status, and self-assessed mouth/teeth condition. This is an important finding for future research aimed at employing at-home, self-screening modalities for cancers among GBM, such as those developed by Ross et al., [33, 43] and Nyitray et al. [44]. Future research should build on this work and explore ways to reach GBM with heightened risk for OPC, such as GBM who use tobacco and drink alcohol at high rates, with OPC self-screening modalities.

While this study was the first of its kind, recruiting an online sample of GBM to research OPC risk factors and perceptions, our study has limitations. First, this analysis was an exploratory, cross-sectional study, and thus causality should not and cannot be inferred. Next, each of the dependent variables were measured with single items, and while no validated and robust scales existed measuring these variables at the time of data collection, such approaches could improve internal validity. Tobacco use was self-reported and not biochemically validated which could have led to misclassification of the outcomes. Furthermore, the measures used in this analysis did not account for cumulative lifetime tobacco use but rather determined tobacco use by combining a threshold of lifetime tobacco use and having used tobacco in the past 30 days. Biochemical validation or different measurement approaches might have increased analysis rigor. Similarly, alcohol was a self-reported measure and also not biochemically validated. The subjective nature of self-reported data on perceived risks may also have biased results. To our knowledge, the AUDIT-C has also never been validated among GBM populations. Biochemical validation or different measurement approaches might have also increased the alcohol analysis rigor as well. Additionally, data missingness influenced some of the variables used in this analysis. We attempted to use statistical approaches to impute missing data; however, these approaches did not benefit analytical modeling. Therefore, we removed missing data from the analysis, which may have biased results. Importantly, we recruited for this study using convenience sampling via online dating apps, so we cannot know the generalizability of the results, and results may not generalize to GBM who are in monogamous relationships. Moreover, while this study was based on random advertising by geography and time of day, it is likely that GBM who already knew of, and were concerned about oropharyngeal cancer, responded to the ads about an oropharyngeal cancer study and completed the questionnaire, and therefore results may be subject to selection bias. Lastly, some gender and sexual orientation categories with low sample sizes were collapsed into larger categories and results may therefore not generalize to those categories that were collapsed.

### Conclusions

Despite increased cancer risk among gay and bisexual people, there are no guidelines for oropharyngeal cancer screening or early detection among this population [38]. Our analysis demonstrated that GBM who experience some OPC risk factors, such as cigarette smoking, dual use of vaping and smoking, higher numbers of sexual partners, and poor self-assessed mouth/teeth condition, also consider themselves at higher risk for OPC. Our analysis also demonstrated that other established OPC risk factors were not associated with increased perceived risk among GBM, importantly hazardous drinking. Furthermore, the results suggest that Hispanic GBM are more worried about what a doctor may find if screened for OPC relative to White men. This implies that OPC screening and early detection efforts should consider ways to reach Hispanic GBM men in ways that emphasize the benefits of screening and that resonate with the needs of this particular community. Lastly, our analysis found that GBM felt comfortable with OPC self-screening regardless of multiple risk and demographic factors. These outcomes provide critical information for future efforts to educate GBM on OPC prevention and early detection. They also provide important considerations for future clinical screening guidelines and self-screening approaches.

## Electronic supplementary material

Below is the link to the electronic supplementary material.

Supplementary Material 1

## Data Availability

The datasets generated and/or analyzed during the current study are not publicly available due because the study is still ongoing.

## Abbreviations

CI: Confidence Interval
GBM: Gay and Bisexual Men
HIV: Human Immunodeficiency Virus
HPV: Human Papillomavirus-16 and − 18
OPC: Oropharyngeal Cancer
