# Supplementary material for: The influence of tobacco use, hazardous drinking, and other risk factors on HPV-associated oropharyngeal cancer risk and screening perceptions among gay and bisexual men: a cross-sectional study
Source: BMC Oral Health. 2025 Mar 30;25:462. doi: 10.1186/s12903-025-05774-0 (PMC11955142; doi:10.1186/s12903-025-05774-0)
Supplement: Supplementary file 1 — Supplementary Material 1 [file 12903_2025_5774_MOESM1_ESM.docx]

Aim 2 ORCA

Start of Block: ES_Eligibility

| 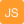 | 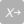 |
| --- | --- |

ES1 What is your gender?

- Male (1)
- Female (0)
- Other (please specify): (-8) ________________________________________________

| Page Break |  |
| --- | --- |

| 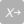 |
| --- |

ES2 In the past 5 years, have you had any type of sex with a man?

- Yes (1)
- No (0)

| Page Break |  |
| --- | --- |

| 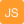 | 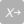 | 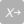 |
| --- | --- | --- |

ES3 What do you identify as?

- Gay (homosexual) (1)
- Bisexual (2)
- Straight (heterosexual) (0)
- Other (please specify): (-8) ________________________________________________

| Page Break |  |
| --- | --- |

| 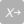 | 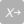 |
| --- | --- |

ES4 What is your current age in years?

- Under the age of 18 years (0)
- 18 years or older (1)

| Page Break |  |
| --- | --- |

| 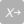 |
| --- |

ES5 Do you **currently** live within one of the 50 United States, Washington D.C., a United States Territory (American Samoa, Guam, Northern Mariana Islands, Puerto Rico, or the U.S. Virgin Islands), or an overseas United States Military address?

- Yes (1)
- No (0)

| Page Break |  |
| --- | --- |

ES6 We have to confirm that you're a real person!

| Page Break |  |
| --- | --- |

| 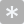 |
| --- |

ES7 Please provide us with your email address so that you can save and return. We will also send you reminders about your survey status, if needed. Your email will remain confidential.

________________________________________________________________

End of Block: ES_Eligibility

Start of Block: Consent Section

Q1 Welcome to the ORCA Study on ***Or***opharyngeal ***Ca***ncer in gay and bisexual men! 

 The next few pages will provide information on the study to help you decide whether or not you would like to participate in this research.

| Page Break |  |
| --- | --- |

Consent: About **About This Research Study** This is the **first** National Institute of Health-funded study on oropharyngeal cancer (of the back of the mouth, tonsils and throat) in gay and bisexual men and men who have sex with men. Oropharyngeal cancer is the 8th most common cancer in U.S. men. However, few studies have looked at oropharyngeal cancer prevention in gay and bisexual men. 
 This is an online survey study that will take about **30-60 minutes** (on average) to complete. You may take breaks as needed and return to the survey. You are only eligible to take the survey *once*. You will only need a device, such as a smartphone or computer, to complete this survey and there will be no cost to you for any of the study activities or procedures. 
 As a thank you, participants who have a complete and valid survey will be sent a $50 Amazon e-gift card within 5-7 business days after finishing the survey.

 **What happens if I do not want to be in this research or change my mind?** There are no alternatives, other than deciding not to participate in this research study. You can leave the research study at any time and no one will be upset by your decision. Your decision whether or not to participate *will not affect* your current or future relations with the University of Minnesota or the National Institutes of Health.
 **Financial Interest Disclosure:** The study team has no financial interests to disclose.
 **Supported By:**This research is supported by the National Institutes of Health and the University of Minnesota (5R01CA253244-02).

| 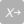 | 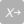 |
| --- | --- |

Consent1 **Would you like to continue?**

- **Yes**, I would like to continue. (1)
- **No**, I would **not** like to continue. (0)

| Page Break |  |
| --- | --- |

Display This Question:

If Would you like to continue? = <strong>Yes</strong>, I would like to continue.

Consent: Survey Info **What is the survey about?** The survey asks questions about what you know and think about oropharyngeal cancer, your sexual behaviors, and risk factors for oropharyngeal cancer. The survey will also ask questions about what screening methods and approaches would be acceptable to you.  We are interested in hearing from many perspectives and invite you to participate *regardless* of your current knowledge about oropharyngeal cancer, healthcare seeking behaviors, sexual practices, or substance use. Please note that we cannot promise any direct benefits to you or others from your taking part in this research.   *We value and appreciate the time you take to contribute to this important research, if you decide to participate.*   **Why am I being invited to take part in this research study?** We are asking you to take part in this research study because you have been selected from Scruff, Jack’d, or Hornet.
 You may be eligible to participate if you meet the following criteria:  • You identify as a man. *We invite you to participate regardless of your sex assigned at birth.* • You identify as a gay or bisexual man, or a man who has sex with men.  • You have had sex with a man in the last five years.  • You are over the age of 18 years.  • You currently live in the U.S

Display This Question:

If Would you like to continue? = <strong>Yes</strong>, I would like to continue.

| 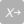 | 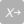 |
| --- | --- |

Consent2 **Would you like to continue?**

- **Yes**, I would like to continue. (1)
- **No**, I would **not** like to continue. (0)

| Page Break |  |
| --- | --- |

Display This Question:

If Would you like to continue? = <strong>Yes</strong>, I would like to continue.

Consent: Risks **Are there any risks?** There are no foreseen risks of research participation. Some of the questions are sexually explicit and some people may find some of the questions embarrassing. We recommend you take the survey in a place where you can answer honestly, but please know you can skip questions you may find uncomfortable.   **Security** This study uses the University of Minnesota’s security standards to protect your information and minimize risks, but there is always a possibility of a data breach. To protect your information, data will be uploaded using encryption and stored behind password protected files.  Only study staff have access to this information. We may be asked to share your information with the University of Minnesota’s Institutional Review Board (IRB), the committee that provides ethical and regulatory oversight of research, and other representatives of this institution, including those that have responsibilities for monitoring or ensuring compliance, if we are audited. Our team works diligently with our online recruiting partners to keep your data secure. This includes monitoring how many people click our ads in their apps.
 Personal identifying information will be stored *separately* from your survey data. Your name and identifying information will be kept confidential and *never* shared in any reports.  **Data Storage** When the study ends, we will store a copy of the survey data (without any identifying information) at the University of Minnesota’s Data Repository. This is to let other researchers check our work and possibly conduct other analyses. All identifiers will be removed from your data, which means that nobody who works with them for future research will know who you are. Therefore, you will not receive any results or financial benefit from future research done on your data.

Display This Question:

If Would you like to continue? = <strong>Yes</strong>, I would like to continue.

| 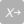 | 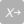 |
| --- | --- |

Consent3 **Would you like to continue?**

- **Yes**, I would like to continue. (1)
- **No**, I would **not** like to continue. (0)

| Page Break |  |
| --- | --- |

Display This Question:

If Would you like to continue? = <strong>Yes</strong>, I would like to continue.

Consent: Feedback **What if I have questions before, during, or after taking the survey?**
   **If you have any questions or concerns, please contact our study team.** **Investigator Team Contact Information:** **Principal Investigator:** Michael W. Ross, PhD MedDr, Family Medicine (Institute for Sexual and Gender Health) at the University of Minnesota. Email: [mwross@umn.edu](mailto:mwross@umn.edu) **Study Coordinator:** [orca@umn.edu](mailto:orca@umn.edu). You may contact the study coordinator if you need the research explained, have questions about the study, research results, or other concerns. **Whom do I contact if I have questions, concerns or feedback about my experience?**  This research has been reviewed and approved by the University of Minnesota's IRB within the Human Research Protections Program (HRPP). To share feedback privately with the HRPP about your research experience, call the Research Participants’ Advocate Line at 612-625-1650 (Toll Free: 1-888-224-8636) or go to z.umn.edu/participants. You are encouraged to contact the HRPP if: Your questions, concerns, or complaints are not being answered by the research team. You cannot reach the research team. You want to talk to someone besides the research team. You have questions about your rights as a research participant. You want to get information or provide input about this research. **Will I have a chance to provide feedback after the study is over?** The HRPP may ask you to complete a survey that asks about your experience as a research participant. You *do not* have to complete the survey if you do not want to. If you do choose to complete the survey, your responses will be anonymous. If you are not asked to complete a survey, but you would like to share feedback, please contact the study team or the HRPP.

Display This Question:

If Would you like to continue? = <strong>Yes</strong>, I would like to continue.

| 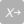 | 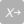 |
| --- | --- |

Consent4 **Would you like to continue?**

- **Yes**, I would like to continue. (1)
- **No**, I would **not** like to continue. (0)

End of Block: Consent Section

Start of Block: A_Demographics

A_INTRO **Great! You're ready to start the survey.

 This first section asks about how you describe yourself.**

| Page Break |  |
| --- | --- |

| 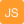 | 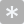 |
| --- | --- |

A01 What is your current age in years?

________________________________________________________________

| Page Break |  |
| --- | --- |

| 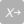 | 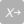 |
| --- | --- |

A02 What sex were you assigned at birth (meaning what is the sex marked on your original birth certificate)?

- Male (1)
- Female (0)
- Intersex (2)
- Prefer not to answer (-9)

| Page Break |  |
| --- | --- |

| 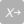 | 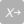 |
| --- | --- |

A03 With which gender identity do you most identify?   *Cisgender: your gender identity matches the sex you were assigned at birth.
 Transgender: your gender identity does not match the sex you were assigned at birth.
 Non-binary, gender non-conforming: your gender identity does not fit inside traditional male or female categories.*

- Cisgender man (1)
- Cisgender woman (0)
- Transgender man (2)
- Transgender woman (3)
- Non-binary, gender non-conforming (4)
- Not listed (please specify): (-8) ________________________________________________
- Prefer not to answer (-9)

| Page Break |  |
| --- | --- |

| 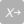 | 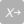 |
| --- | --- |

A04 Do you think of yourself as:

- Gay (or homosexual) (1)
- Bisexual (2)
- Straight (or heterosexual) (0)
- Not listed (please specify): (-8) ________________________________________________
- Prefer not to answer (-9)

| Page Break |  |
| --- | --- |

| 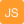 | 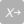 | 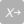 |
| --- | --- | --- |

A05 What is your current relationship status?

- Single (0)
- Dating, **not** living with a partner (1)
- Dating, living with a partner (2)
- Married or civil union (3)
- Separated (4)
- Divorced (5)
- Widowed (6)
- Not listed (please specify): (-8) ________________________________________________
- Prefer not to answer (-9)

| Page Break |  |
| --- | --- |

| 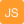 | 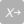 | 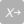 |
| --- | --- | --- |

A06 What is your race? *(Check all that apply)*

- American Indian or Alaska Native (1)
- Asian (2)
- Black or African American (3)
- Native Hawaiian or other Pacific Islander (4)
- White (0)
- Not listed (please specify): (-8) ________________________________________________
- ⊗Prefer not to answer (-9)

| Page Break |  |
| --- | --- |

| 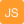 | 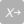 | 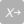 |
| --- | --- | --- |

A07 Are you Spanish/Hispanic/Latino? *(Check all that apply)*

- ⊗**No**, not of Spanish, Hispanic, or Latino origin (0)
- **Yes**, Mexican, Mexican American, Chicano (1)
- **Yes**, Puerto Rican (2)
- **Yes**, Cuban (3)
- **Yes**, another Spanish, Hispanic, or Latino origin not listed (please specify): (-8) ________________________________________________
- ⊗Prefer not to answer (-9)

| Page Break |  |
| --- | --- |

| 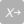 | 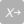 |
| --- | --- |

A08 What is the highest level of education you have completed?

- Less than high school (1)
- High school graduate or GED (0)
- Some college, but no degree (2)
- Associate's degree (3)
- Bachelor's degree (4)
- Graduate or professional degree (5)
- Prefer not to answer (-9)

| Page Break |  |
| --- | --- |

| 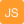 | 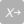 | 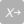 |
| --- | --- | --- |

A09 Who do you currently live with? *(Check all that apply)*

- Partner/spouse (0)
- Friends or roommates (1)
- Adult family members (parents, siblings, adult children) (2)
- Family members under the age of 18 (children, siblings, grandchildren, niece/nephew) (3)
- Not listed (please specify): (-8) ________________________________________________
- ⊗Not applicable, I live alone (4)
- ⊗Prefer not to answer (-9)

Skip To: A10 If Who do you currently live with? (Check all that apply) = Not applicable, I live alone

Skip To: A10 If Who do you currently live with? (Check all that apply) = Prefer not to answer

| Page Break |  |
| --- | --- |

Display This Question:

If Who do you currently live with? (Check all that apply) = Partner/spouse

Or Who do you currently live with? (Check all that apply) = Friends or roommates

Or Who do you currently live with? (Check all that apply) = Adult family members (parents, siblings, adult children)

Or Who do you currently live with? (Check all that apply) = Family members under the age of 18 (children, siblings, grandchildren, niece/nephew)

Or Or Who do you currently live with? (Check all that apply) Text Response Is Not Empty

| 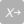 | 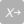 |
| --- | --- |

A09_b How many people, other than yourself, live in your household? *(If you live in a multi-unit building, only count those in your unit)*

▼ 0 (0) ... Prefer not to answer (-9)

| Page Break |  |
| --- | --- |

Display This Question:

If How many people, other than yourself, live in your household? (If you live in a multi-unit buildi... = 1

Or How many people, other than yourself, live in your household? (If you live in a multi-unit buildi... = 2

Or How many people, other than yourself, live in your household? (If you live in a multi-unit buildi... = 3

Or How many people, other than yourself, live in your household? (If you live in a multi-unit buildi... = 4

Or How many people, other than yourself, live in your household? (If you live in a multi-unit buildi... = 5

Or How many people, other than yourself, live in your household? (If you live in a multi-unit buildi... = 6

Or How many people, other than yourself, live in your household? (If you live in a multi-unit buildi... = 7

Or How many people, other than yourself, live in your household? (If you live in a multi-unit buildi... = 8

Or How many people, other than yourself, live in your household? (If you live in a multi-unit buildi... = 9

Or How many people, other than yourself, live in your household? (If you live in a multi-unit buildi... = 10

Or How many people, other than yourself, live in your household? (If you live in a multi-unit buildi... = 11

Or How many people, other than yourself, live in your household? (If you live in a multi-unit buildi... = 12

Or How many people, other than yourself, live in your household? (If you live in a multi-unit buildi... = 13

Or How many people, other than yourself, live in your household? (If you live in a multi-unit buildi... = 14

Or How many people, other than yourself, live in your household? (If you live in a multi-unit buildi... = 15

Or How many people, other than yourself, live in your household? (If you live in a multi-unit buildi... = 16

Or How many people, other than yourself, live in your household? (If you live in a multi-unit buildi... = 17

Or How many people, other than yourself, live in your household? (If you live in a multi-unit buildi... = 18

Or How many people, other than yourself, live in your household? (If you live in a multi-unit buildi... = 19

Or How many people, other than yourself, live in your household? (If you live in a multi-unit buildi... = 20

| 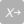 | 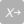 |
| --- | --- |

A09_c How many of the people in your household do you claim as dependents?

▼ 0 (0) ... Prefer not to answer (-9)

| Page Break |  |
| --- | --- |

Display This Question:

If How many of the people in your household do you claim as dependents? = 1

Or How many of the people in your household do you claim as dependents? = 2

Or How many of the people in your household do you claim as dependents? = 3

Or How many of the people in your household do you claim as dependents? = 4

Or How many of the people in your household do you claim as dependents? = 5

Or How many of the people in your household do you claim as dependents? = 6

Or How many of the people in your household do you claim as dependents? = 7

Or How many of the people in your household do you claim as dependents? = 8

Or How many of the people in your household do you claim as dependents? = 9

Or How many of the people in your household do you claim as dependents? = 10

Or How many of the people in your household do you claim as dependents? = 11

Or How many of the people in your household do you claim as dependents? = 12

Or How many of the people in your household do you claim as dependents? = 13

Or How many of the people in your household do you claim as dependents? = 14

Or How many of the people in your household do you claim as dependents? = 15

Or How many of the people in your household do you claim as dependents? = 16

Or How many of the people in your household do you claim as dependents? = 17

Or How many of the people in your household do you claim as dependents? = 18

Or How many of the people in your household do you claim as dependents? = 19

Or How many of the people in your household do you claim as dependents? = 20

| 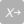 | 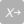 |
| --- | --- |

A09_d How many of your dependents are under the age of 18?

▼ 0 (0) ... Prefer not to answer (-9)

| Page Break |  |
| --- | --- |

A10 What zip code do you currently live in?

- Please enter your 5-digit zip code: (1) ________________________________________________

End of Block: A_Demographics

Start of Block: B_Healthcare

B_INTRO **This next section will ask about your experiences accessing healthcare.**

| Page Break |  |
| --- | --- |

| 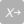 | 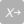 |
| --- | --- |

B01 **Before starting this survey**, how often have you **heard** about oropharyngeal cancer?

- Never (this is my first time hearing about it) (0)
- Rarely (only once or twice before now) (1)
- Sometimes (a few times a year) (2)
- Often (about once a month) (3)
- Always (almost every day) (4)
- Prefer not to answer (-9)

| Page Break |  |
| --- | --- |

| 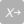 | 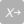 |
| --- | --- |

B02 **Before starting this survey**, have you ever thought about asking a healthcare provider (like a doctor or dentist) to check you for oropharyngeal cancer?

- Yes (1)
- No (0)
- Prefer not to answer (-9)

| Page Break |  |
| --- | --- |

| 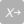 | 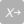 |
| --- | --- |

B03 Do you have a regular **doctor,** primary care physician, physician assistant, or nurse that you see?
 *(This would be a provider that you see for regular check-ups)*

- Yes (1)
- No (0)
- Prefer not to answer (-9)

| Page Break |  |
| --- | --- |

| 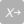 | 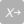 |
| --- | --- |

B04 Do you have a regular **dentist** that you see?
 *(This would be a dentist you see for regular dental check-ups)*

- Yes (1)
- No (0)
- Prefer not to answer (-9)

| Page Break |  |
| --- | --- |

| 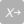 | 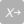 |
| --- | --- |

B05 Have you received a **COVID-19 vaccination**? *(Common names include Pfizer, Moderna, or Johnson&Johnson)*

- Yes (1)
- No (0)
- Prefer not to answer (-9)

| Page Break |  |
| --- | --- |

Display This Question:

If Have you received a COVID-19 vaccination? (Common names include Pfizer, Moderna, or Johnson&Johnson) = No

| 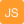 | 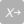 | 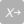 |
| --- | --- | --- |

B05_b What is the **main reason** you have **not** received a **COVID-19 vaccination**?

- I haven't had time (i.e. I can't get time off work, other priorities) (0)
- I have already had COVID-19 so I don't believe a vaccine is necessary (1)
- I would rather take the risk of getting sick with COVID-19 (2)
- COVID-19 is not a big deal, it’s like the flu (3)
- I’m worried about COVID-19 vaccine safety or side effects (4)
- I don't like injections (5)
- I don’t know how to schedule an appointment to get the COVID-19 vaccine (6)
- I don't know where to get vaccinated (7)
- I don’t want to make multiple appointments to finish the COVID-19 vaccine series (8)
- I don’t have transportation to get the COVID-19 vaccine (9)
- I don’t trust what pharmaceutical companies put in the COVID-19 vaccine (10)
- I distrust the U.S. healthcare system (11)
- I distrust the government’s role in vaccine distribution (12)
- Not listed (please specify): (-8) ________________________________________________
- Prefer not to answer (-9)

| Page Break |  |
| --- | --- |

| 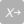 | 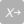 |
| --- | --- |

B06 Have you received at least one dose of the **human papillomavirus (HPV) vaccine**?
 *(Common names include Gardasil and Cervarix)*

 HPV: human papillomavirus is a virus that can be sexually transmitted

- Yes (1)
- No (0)
- Don't know/Not sure (2)
- Prefer not to answer (-9)

Display This Question:

If Have you received at least one dose of the human papillomavirus (HPV) vaccine?(Common names inclu... = Yes

| 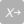 | 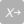 |
| --- | --- |

B06_bb How many doses of the **HPV vaccination series** have you received?

- 1 dose (1)
- 2 doses (2)
- 3 doses (3)
- Don't know/Not sure (4)

| Page Break |  |
| --- | --- |

Display This Question:

If Have you received at least one dose of the human papillomavirus (HPV) vaccine?(Common names inclu... = No

| 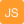 | 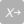 | 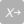 |
| --- | --- | --- |

B06_c What is the **main reason** you have **not** received the **HPV vaccine**?

- A doctor never recommended or offered me the HPV vaccine (1)
- The vaccine was not available to me (due to age or gender restrictions) (2)
- I’ve never heard of the HPV vaccine (3)
- My parents, guardians, or family members wouldn’t let me get the HPV vaccine (4)
- I haven’t had time (i.e. I can’t get time off work, forgot, other priorities) (0)
- Cost of the HPV vaccine (including lack of insurance) (5)
- The HPV vaccine is just for girls and women (7)
- The HPV vaccine is just for straight (heterosexual) people (8)
- The HPV vaccine is more effective for girls than for boys (9)
- I don’t like injections (10)
- I don’t know where to get the HPV vaccine (11)
- I don’t know how to schedule an appointment to get the HPV vaccine (12)
- I don’t want to make multiple appointments to finish the HPV vaccine series (13)
- I don’t have transportation to get the HPV vaccine (14)
- I distrust the U.S. healthcare system (15)
- I don’t trust what pharmaceutical companies put in the HPV vaccine (16)
- I have concerns about HPV vaccine safety (17)
- HPV is just another sexually transmitted infection (STI), it’s not a big deal (18)
- Not listed (please specify): (-8) ________________________________________________
- Prefer not to answer (-9)

| Page Break |  |
| --- | --- |

Display This Question:

If Have you received at least one dose of the human papillomavirus (HPV) vaccine?(Common names inclu... = No

Or Have you received at least one dose of the human papillomavirus (HPV) vaccine?(Common names inclu... = Don't know/Not sure

Or Have you received at least one dose of the human papillomavirus (HPV) vaccine?(Common names inclu... = Prefer not to answer

B_INTRO2 **Transitioning to a few questions about your opinions on the HPV vaccine.**

| Page Break |  |
| --- | --- |

Display This Question:

If Have you received at least one dose of the human papillomavirus (HPV) vaccine?(Common names inclu... = No

Or Have you received at least one dose of the human papillomavirus (HPV) vaccine?(Common names inclu... = Don't know/Not sure

Or Have you received at least one dose of the human papillomavirus (HPV) vaccine?(Common names inclu... = Prefer not to answer

| 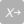 | 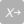 |
| --- | --- |

B07 I would get the HPV vaccine to prevent oropharyngeal cancer even if I had to pay around $350 out of pocket.

 HPV: human papillomavirus is a virus that can be sexually transmitted

- Strongly agree (4)
- Somewhat agree (3)
- Neither agree nor disagree (2)
- Somewhat disagree (1)
- Strongly disagree (0)
- Prefer not to answer (-9)

| Page Break |  |
| --- | --- |

Display This Question:

If Have you received at least one dose of the human papillomavirus (HPV) vaccine?(Common names inclu... = No

Or Have you received at least one dose of the human papillomavirus (HPV) vaccine?(Common names inclu... = Don't know/Not sure

Or Have you received at least one dose of the human papillomavirus (HPV) vaccine?(Common names inclu... = Prefer not to answer

| 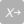 | 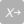 |
| --- | --- |

B08 I am willing to discuss the HPV vaccine with **a doctor.**

 HPV: human papillomarivus is a virus that can be sexually transmitted

- Strongly agree (4)
- Somewhat agree (3)
- Neither agree nor disagree (2)
- Somewhat disagree (1)
- Strongly disagree (0)
- Prefer not to answer (-9)

| Page Break |  |
| --- | --- |

Display This Question:

If Have you received at least one dose of the human papillomavirus (HPV) vaccine?(Common names inclu... = No

Or Have you received at least one dose of the human papillomavirus (HPV) vaccine?(Common names inclu... = Don't know/Not sure

Or Have you received at least one dose of the human papillomavirus (HPV) vaccine?(Common names inclu... = Prefer not to answer

| 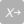 | 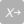 |
| --- | --- |

B09 I intend to make an appointment with a healthcare provider (like a doctor or pharmacist) to get the **HPV vaccine** in the next 30 days.

 HPV: human papillomavirus is a virus that is sexually transmitted

- Strongly agree (4)
- Somewhat agree (3)
- Neither agree nor disagree (2)
- Somewhat disagree (1)
- Strongly disagree (0)
- Prefer not to answer (-9)

End of Block: B_Healthcare

Start of Block: C_Oral Health

C_INTRO **Great work so far! Next, we're going to ask questions about your oral health.**

| Page Break |  |
| --- | --- |

| 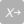 | 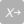 |
| --- | --- |

C01 How would you describe the condition of your mouth and teeth?

- Poor (3)
- Fair (2)
- Good (1)
- Very good (0)
- Prefer not to answer (-9)

| Page Break |  |
| --- | --- |

| 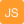 | 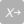 | 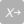 |
| --- | --- | --- |

C02 How often have you experienced each of the following problems **related to your mouth and teeth** during the past 12 months?

|  | Never (0) | Rarely (1) | Occasionally (2) | Very Often (3) | Prefer not to answer (-9) |
| --- | --- | --- | --- | --- | --- |
| Difficulty when biting or chewing foods (1) |  |  |  |  |  |
| Difficulty with speech or pronouncing words (2) |  |  |  |  |  |
| Dry mouth (3) |  |  |  |  |  |
| Felt anxiety (related to your mouth or teeth) (4) |  |  |  |  |  |
| Felt embarrassment (5) |  |  |  |  |  |
| Avoided smiling (6) |  |  |  |  |  |
| Took days off work because of pain or discomfort (7) |  |  |  |  |  |
| Difficulty doing usual activities (8) |  |  |  |  |  |
| Reduced participation in social activities (9) |  |  |  |  |  |
| Problems sleeping (10) |  |  |  |  |  |
| Experienced pain (11) |  |  |  |  |  |

| Page Break |  |
| --- | --- |

| 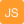 | 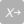 | 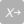 |
| --- | --- | --- |

C03 How strongly do you agree or disagree with the following statements about how **you perceive the health of your mouth**?

|  | Strongly agree (4) | Somewhat agree (3) | Neither agree nor disagree (2) | Somewhat disagree (1) | Strongly disagree (0) | Prefer not to answer (-9) |
| --- | --- | --- | --- | --- | --- | --- |
| I value keeping my mouth healthy (1) |  |  |  |  |  |  |
| Regular visits to the dentist will help keep me healthy (2) |  |  |  |  |  |  |
| As I grow old I accept that I will lose some of my teeth (3) |  |  |  |  |  |  |
| I need to see the dentist at least twice a year (4) |  |  |  |  |  |  |
| It is easier to get ahead in life if I have good teeth (5) |  |  |  |  |  |  |

| Page Break |  |
| --- | --- |

| 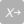 | 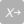 |
| --- | --- |

C04 How long has it been since you last had a dental visit?

- Less than 12 months (0)
- 1 to 2 years (1)
- 3 to 5 years (2)
- More than 5 years (3)
- Not applicable-I have never been to a dentist (-7)
- Prefer not to answer (-9)

| Page Break |  |
| --- | --- |

Display This Question:

If How long has it been since you last had a dental visit? != Less than 12 months

| 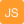 | 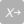 | 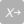 |
| --- | --- | --- |

C04_b Why did you **not** visit the dentist more frequently? *(Check all that apply)*

- My mouth is healthy so I do not need to visit the dentist (0)
- I do not know where to go to receive dental services (1)
- I cannot afford to go to the dentist (2)
- I did not go due to the COVID-19 pandemic (3)
- It is too hard to find a dentist that accepts my dental plan or insurance (4)
- I cannot find the time to get to a dentist (i.e., cannot get time off from work, dentist does not have convenient clinic hours) (5)
- Many services are not covered by my dental plan or insurance, so I end up having to pay with my own money (6)
- I cannot travel to a dentist easily (i.e., do not have transportation, clinic is located too far away) (7)
- I do not have any of my original teeth (i.e., I have no teeth or I have dentures) (8)
- I am afraid of going to the dentist (9)
- Not listed (please specify): (-8) ________________________________________________
- ⊗No particular reason (10)

| Page Break |  |
| --- | --- |

| 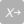 | 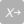 |
| --- | --- |

C05 In the last 12 months, have you gone to an emergency dentist or emergency room **for tooth or mouth pain** (*not* related to an injury)?

- Yes (1)
- No (0)
- Prefer not to answer (-9)

| Page Break |  |
| --- | --- |

| 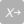 | 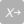 |
| --- | --- |

C06 Do you plan to visit a non-emergency dentist in the next 12 months?

- Yes (1)
- No (0)
- Prefer not to answer (-9)

End of Block: C_Oral Health

Start of Block: D_Knowledge

D_INTRO **This section will ask questions about what you currently know about oropharyngeal cancer. Please answer honestly, it is okay if you don't know anything!**
 You are 1/3 of the way through the survey! Remember, you can save and return later if want to take a break.

| Page Break |  |
| --- | --- |

| 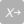 | 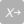 |
| --- | --- |

D01 Oropharyngeal cancer is the same as oral (mouth) cancer.

- True (1)
- False (0)
- Don't know/Not sure (2)
- Prefer not to answer (-9)

| Page Break |  |
| --- | --- |

| 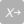 | 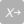 |
| --- | --- |

D02 Oropharyngeal cancer includes cancer of the back of the tongue and the tonsils.

- True (1)
- False (0)
- Don't know/Not sure (2)
- Prefer not to answer (-9)

| Page Break |  |
| --- | --- |

| 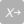 | 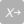 |
| --- | --- |

D03 Dentists cannot check for oropharyngeal cancer.

- True (1)
- False (0)
- Don't know/Not sure (2)
- Prefer not to answer (-9)

| Page Break |  |
| --- | --- |

| 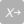 | 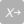 |
| --- | --- |

D04 There are two main types of oropharyngeal cancer, one caused by HPV-16, the other caused by heavy drinking and smoking.   HPV: human papillomavirus is a virus that can be sexually transmitted

- True (1)
- False (0)
- Don't know/Not sure (2)
- Prefer not to answer (-9)

| Page Break |  |
| --- | --- |

| 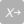 | 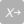 |
| --- | --- |

D05 Young people of all genders can be vaccinated against HPV-related cancers.
   HPV: human papillomavirus is a virus that can be sexually transmitted

- True (1)
- False (0)
- Don't know/Not sure (2)
- Prefer not to answer (-9)

| Page Break |  |
| --- | --- |

D_INTRO2 Great job! We will have a few more knowledge questions towards the end of the survey.

End of Block: D_Knowledge

Start of Block: E_Attitudes

E_INTRO **The following questions will ask about your opinions towards different health topics.**

| Page Break |  |
| --- | --- |

| 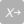 |  |
| --- | --- |

E01 I believe I have several risk factors for oropharyngeal cancer.

- Strongly agree (1)
- Somewhat agree (2)
- Neither agree nor disagree (3)
- Somewhat disagree (4)
- Strongly disagree (5)
- Prefer not to answer (-9)

| Page Break |  |
| --- | --- |

|  |  |
| --- | --- |

E02 It is worthwhile checking for oropharyngeal cancer.

- Strongly agree (4)
- Somewhat agree (3)
- Neither agree nor disagree (2)
- Somewhat disagree (1)
- Strongly disagree (0)
- Prefer not to answer (-9)

| Page Break |  |
| --- | --- |

|  |  |
| --- | --- |

E03 There are so many health hazards out there it is too exhausting to consider them all.

- Strongly agree (4)
- Somewhat agree (3)
- Neither agree nor disagree (2)
- Somewhat disagree (1)
- Strongly disagree (0)
- Prefer not to answer (-9)

| Page Break |  |
| --- | --- |

|  |  |
| --- | --- |

E04 People are just trying to attack gay and bisexual men by frightening them about sexual health risks, like oropharyngeal cancer.

- Strongly agree (4)
- Somewhat agree (3)
- Neither agree nor disagree (2)
- Somewhat disagree (1)
- Strongly disagree (0)
- Prefer not to answer (-9)

| Page Break |  |
| --- | --- |

E_INTRO2 **Next, we have a few questions about your doctor or dentist. This can be your regular doctor or dentist or how you would feel if you were to see a doctor or dentist.**

| Page Break |  |
| --- | --- |

|  |  |
| --- | --- |

E05 I would feel comfortable telling **a doctor** about my sexual history as part of a routine cancer screening.

- Strongly agree (4)
- Somewhat agree (3)
- Neither agree nor disagree (2)
- Somewhat disagree (1)
- Strongly disagree (0)
- Prefer not to answer (-9)

| Page Break |  |
| --- | --- |

|  |  |
| --- | --- |

E06 I feel nervous when I visit **a doctor** for routine care.

- Strongly agree (4)
- Somewhat agree (3)
- Neither agree nor disagree (2)
- Somewhat disagree (1)
- Strongly disagree (0)
- Prefer not to answer (-9)

| Page Break |  |
| --- | --- |

|  |  |
| --- | --- |

E07 I can afford to see **a doctor** for routine care.

- Strongly agree (4)
- Somewhat agree (3)
- Neither agree nor disagree (2)
- Somewhat disagree (1)
- Strongly disagree (0)
- Prefer not to answer (-9)

| Page Break |  |
| --- | --- |

|  |  |
| --- | --- |

E08 I would feel comfortable telling **a dentist** about my sexual history as part of a routine cancer screening.

- Strongly agree (4)
- Somewhat agree (3)
- Neither agree nor disagree (2)
- Somewhat disagree (1)
- Strongly disagree (0)
- Prefer not to answer (-9)

| Page Break |  |
| --- | --- |

|  |  |
| --- | --- |

E09 I feel nervous when I visit **a dentist** for routine care.

- Strongly agree (4)
- Somewhat agree (3)
- Neither agree nor disagree (2)
- Somewhat disagree (1)
- Strongly disagree (0)
- Prefer not to answer (-9)

| Page Break |  |
| --- | --- |

|  |  |
| --- | --- |

E10 I can afford to see **a dentist** for routine care.

- Strongly agree (4)
- Somewhat agree (3)
- Neither agree nor disagree (2)
- Somewhat disagree (1)
- Strongly disagree (0)
- Prefer not to answer (-9)

| Page Break |  |
| --- | --- |

|  |  |
| --- | --- |

E11 I'm scared of what a healthcare provider (like a doctor or a dentist) might find if I get screened for oropharyngeal cancer.

- Strongly agree (4)
- Somewhat agree (3)
- Neither agree nor disagree (2)
- Somewhat disagree (1)
- Strongly disagree (0)
- Prefer not to answer (-9)

| Page Break |  |
| --- | --- |

|  |  |
| --- | --- |

E12 Oropharyngeal cancer screening involves someone looking in the mouth and throat for signs of cancer. I would find oropharyngeal cancer screening too invasive.

- Strongly agree (4)
- Somewhat agree (3)
- Neither agree nor disagree (2)
- Somewhat disagree (1)
- Strongly disagree (0)
- Prefer not to answer (-9)

| Page Break |  |
| --- | --- |

|  |  |
| --- | --- |

E13 I would feel comfortable getting checked for oropharyngeal cancer if I could do a self-screening at home.

- Strongly agree (4)
- Somewhat agree (3)
- Neither agree nor disagree (2)
- Somewhat disagree (1)
- Strongly disagree (0)
- Prefer not to answer (-9)

| Page Break |  |
| --- | --- |

|  |  |
| --- | --- |

E14 I would be influenced to do what my friends say about screening for oropharyngeal cancer.

- Strongly agree (4)
- Somewhat agree (3)
- Neither agree nor disagree (2)
- Somewhat disagree (1)
- Strongly disagree (0)
- Prefer not to answer (-9)

| Page Break |  |
| --- | --- |

|  |  |
| --- | --- |

E15 I worry about the cost of screening for oropharyngeal cancer.

- Strongly agree (4)
- Somewhat agree (3)
- Neither agree nor disagree (2)
- Somewhat disagree (1)
- Strongly disagree (0)
- Prefer not to answer (-9)

| Page Break |  |
| --- | --- |

|  |  |
| --- | --- |

E16 I do not think that any vaccinations are safe.

- Strongly agree (4)
- Somewhat agree (3)
- Neither agree nor disagree (2)
- Somewhat disagree (1)
- Strongly disagree (0)
- Prefer not to answer (-9)

End of Block: E_Attitudes

Start of Block: F_Beliefs

F_INTRO **This section will ask questions about your thoughts towards oropharyngeal cancer.**

| Page Break |  |
| --- | --- |

|  |  |
| --- | --- |

F01 I believe the benefits of the HPV vaccine outweigh the potentials risks of the vaccine.

 HPV: human papillomavirus is a virus that is sexually transmitted

- Strongly agree (4)
- Somewhat agree (3)
- Neither agree nor disagree (2)
- Somewhat disagree (1)
- Strongly disagree (0)
- Prefer not to answer (-9)

| Page Break |  |
| --- | --- |

|  |  |
| --- | --- |

F02 In general, I believe that vaccines do a good job preventing the diseases that they are meant to prevent.

- Strongly agree (4)
- Somewhat agree (3)
- Neither agree nor disagree (2)
- Somewhat disagree (1)
- Strongly disagree (0)
- Prefer not to answer (-9)

| Page Break |  |
| --- | --- |

|  |  |
| --- | --- |

F03 Compared to the average person, I believe **my risk** of getting oropharyngeal cancer is:

- Much higher (4)
- Somewhat higher (3)
- About the same (2)
- Somewhat lower (1)
- Much lower (0)
- Prefer not to answer (-9)

| Page Break |  |
| --- | --- |

|  |  |
| --- | --- |

F04 Compared to other cancers, I believe that **the severity**of oropharyngeal cancer is:

- Much higher (4)
- Somewhat higher (3)
- About the same (2)
- Somewhat lower (1)
- Much lower (0)
- Prefer not to answer (-9)

| Page Break |  |
| --- | --- |

|  |  |
| --- | --- |

F05 Compared to other early cancer screenings, I believe that **the benefits**of checking for oropharyngeal cancer early are:

- Much higher (4)
- Somewhat higher (3)
- About the same (2)
- Somewhat lower (1)
- Much lower (0)
- Prefer not to answer (-9)

| Page Break |  |
| --- | --- |

|  |  |
| --- | --- |

F06 I believe it will be easy for me to get screened for oropharyngeal cancer.

- Strongly agree (4)
- Somewhat agree (3)
- Neither agree nor disagree (2)
- Somewhat disagree (1)
- Strongly disagree (0)
- Prefer not to answer (-9)

End of Block: F_Beliefs

Start of Block: G_Tobacco

G_INTRO **Please keep going! This section will ask about your previous and current use of tobacco products.**
 
We value the time you are taking to respond to our survey so far. If you need to take a break you can save and return later.

| Page Break |  |
| --- | --- |

|  |  |  |
| --- | --- | --- |

G01 **In your lifetime**, have you ever tried the following tobacco products, **even just one time**? Please do not include marijuana use.
 *(Check all that apply)*

- Cigarettes (0)
- Cigars, cigarillos, or filtered cigars (1)
- Pipes (2)
- Hookah (3)
- Smokeless tobacco (dip, spit, moist snuff, pouches, and chewing tobacco) (4)
- Snus (5)
- Electronic nicotine products (vapes, e-cigarettes, e-cigars, or similar) (6)
- Not listed (please specify): (-8) ________________________________________________
- ⊗None, I have never tried a tobacco product (7)
- ⊗Prefer not to answer (-9)

Skip To: End of Block If In your lifetime, have you ever tried the following tobacco products, even just one time? Please... = Prefer not to answer

Skip To: End of Block If In your lifetime, have you ever tried the following tobacco products, even just one time? Please... = None, I have never tried a tobacco product

| Page Break |  |
| --- | --- |

Display This Question:

If In your lifetime, have you ever tried the following tobacco products, even just one time? Please... = Cigarettes

G_INTRO_CIGARETTES **These questions will ask about your cigarette use.**

| Page Break |  |
| --- | --- |

Display This Question:

If In your lifetime, have you ever tried the following tobacco products, even just one time? Please... = Cigarettes

|  |  |
| --- | --- |

G02 Have you smoked 100 cigarettes (5 packs) or more in your **entire life**?

- Yes (1)
- No (0)
- Prefer not to answer (-9)

| Page Break |  |
| --- | --- |

Display This Question:

If In your lifetime, have you ever tried the following tobacco products, even just one time? Please... = Cigarettes

And Have you smoked 100 cigarettes (5 packs) or more in your entire life? = Yes

|  |  |
| --- | --- |

G03 Have you smoked a cigarette in the**past 30 days**?

- Yes (1)
- No (0)
- Prefer not to answer (-9)

| Page Break |  |
| --- | --- |

Display This Question:

If Have you smoked a cigarette in the past 30 days? = Yes

|  |  |  |  |
| --- | --- | --- | --- |

G03_b On how many of the **past 30 days** did you smoke cigarettes?

- Enter number of **days** (1) ________________________________________________
- Prefer not to answer (-9)

| Page Break |  |
| --- | --- |

Display This Question:

If Have you smoked a cigarette in the past 30 days? = Yes

|  |  |  |  |
| --- | --- | --- | --- |

G03_c On average, on those days you smoked, how many cigarettes did you usually smoke each day? A pack usually has 20 cigarettes in it.

- Enter number of **cigarettes per day** (1) ________________________________________________
- Prefer not to answer (-9)

| Page Break |  |
| --- | --- |

Display This Question:

If Have you smoked a cigarette in the past 30 days? = Yes

|  |  |  |  |
| --- | --- | --- | --- |

G03_d How many **years** have you smoked cigarettes? Please do not count any time you may have stayed off cigarettes.
 *If you have smoked less than 1 year, please enter 0.*

- Number of **years** (1) ________________________________________________
- Prefer not to answer (-9)

| Page Break |  |
| --- | --- |

Display This Question:

If Have you smoked a cigarette in the past 30 days? = No

|  |  |
| --- | --- |

G04 **As of now**, have you been abstinent from smoking cigarettes for at least 30 days, and intend to completely quit?

- Yes (1)
- No (0)
- Prefer not to answer (-9)

| Page Break |  |
| --- | --- |

Display This Question:

If As of now, have you been abstinent from smoking cigarettes for at least 30 days, and intend to co... = Yes

|  |  |  |  |
| --- | --- | --- | --- |

G04_b About how long has it been since you **last smoked** a cigarette? *If you quit smoking less than 1 year ago, please enter 0.*

- Enter time in **years** (1) ________________________________________________
- Prefer not to answer (-9)

| Page Break |  |
| --- | --- |

Display This Question:

If As of now, have you been abstinent from smoking cigarettes for at least 30 days, and intend to co... = Yes

|  |  |  |  |
| --- | --- | --- | --- |

G05_c How many **years** did you smoke cigarettes? Please do not count any time you may have stayed off cigarettes.
 *If you smoked less than 1 year, please enter 0.*

- Number of **years** (1) ________________________________________________
- Prefer not to answer (-9)

| Page Break |  |
| --- | --- |

Display This Question:

If As of now, have you been abstinent from smoking cigarettes for at least 30 days, and intend to co... = Yes

|  |  |  |  |
| --- | --- | --- | --- |

G05_d Think back to when you smoked cigarettes. On average, on those days you smoked, how many cigarettes did you usually smoke each day? A pack usually has 20 cigarettes in it.

- Enter number of **cigarettes per day** (1) ________________________________________________
- Prefer not to answer (-9)

| Page Break |  |
| --- | --- |

Display This Question:

If In your lifetime, have you ever tried the following tobacco products, even just one time? Please... = Cigars, cigarillos, or filtered cigars

G_INTRO_CIGARS **These questions will ask about your cigar, cigarillo, or filtered cigar use.**

| Page Break |  |
| --- | --- |

Display This Question:

If In your lifetime, have you ever tried the following tobacco products, even just one time? Please... = Cigars, cigarillos, or filtered cigars

|  |  |
| --- | --- |

G06 Have you smoked 100 or more cigars, cigarillos, or filtered cigars in your **entire life**?

- Yes (1)
- No (0)
- Prefer not to answer (-9)

| Page Break |  |
| --- | --- |

Display This Question:

If In your lifetime, have you ever tried the following tobacco products, even just one time? Please... = Cigars, cigarillos, or filtered cigars

And Have you smoked 100 or more cigars, cigarillos, or filtered cigars in your entire life? = Yes

|  |  |
| --- | --- |

G07 Have you smoked a cigar, cigarillo, or filtered cigar in the**past 30 days**?

- Yes (1)
- No (0)
- Prefer not to answer (-9)

| Page Break |  |
| --- | --- |

Display This Question:

If Have you smoked a cigar, cigarillo, or filtered cigar in the past 30 days? = Yes

|  |  |  |  |
| --- | --- | --- | --- |

G07_b On how many of the **past 30 days** did you smoke cigars, cigarillos, or filtered cigars?

- Enter number of **days** (1) ________________________________________________
- Prefer not to answer (-9)

| Page Break |  |
| --- | --- |

Display This Question:

If Have you smoked a cigar, cigarillo, or filtered cigar in the past 30 days? = Yes

|  |  |  |  |
| --- | --- | --- | --- |

G07_c On average, on those days you smoked, how many cigars, cigarillos, or filtered cigars did you usually smoke each day?

- Enter number of **cigars, cigarillos, or filtered cigars per day** (1) ________________________________________________
- Prefer not to answer (-9)

| Page Break |  |
| --- | --- |

Display This Question:

If Have you smoked a cigar, cigarillo, or filtered cigar in the past 30 days? = Yes

|  |  |  |  |
| --- | --- | --- | --- |

G07_d How many **years** have you smoked cigars, cigarillos, or filtered cigars? Please **do not** count any time you may have stayed off smoking cigars, cigarillos, or filtered cigars.
 *If you have smoked less than 1 year, please enter 0.*

- Number of **years** (1) ________________________________________________
- Prefer not to answer (-9)

| Page Break |  |
| --- | --- |

Display This Question:

If Have you smoked a cigar, cigarillo, or filtered cigar in the past 30 days? = No

|  |  |
| --- | --- |

G08 **As of now**, have you been abstinent from smoking cigars, cigarillos, or filtered cigars for at least 30 days, and intend to completely quit?

- Yes (1)
- No (0)
- Prefer not to answer (-9)

| Page Break |  |
| --- | --- |

Display This Question:

If As of now, have you been abstinent from smoking cigars, cigarillos, or filtered cigars for at lea... = Yes

|  |  |  |  |
| --- | --- | --- | --- |

G08_b About how long has it been since you **last smoked** cigars, cigarillos, or filtered cigars?
 *If you quit less than 1 year ago, please enter 0.*

- Enter number of **years** (1) ________________________________________________
- Prefer not to answer (-9)

| Page Break |  |
| --- | --- |

Display This Question:

If As of now, have you been abstinent from smoking cigars, cigarillos, or filtered cigars for at lea... = Yes

|  |  |  |  |
| --- | --- | --- | --- |

G08_c How many **years** did you smoke cigars, cigarillos, or filtered cigars? Please **do not** count any time you may have stayed off smoking cigars, cigarillos, or filtered cigars.
 *If you smoked less than 1 year, please enter 0.*

- Number of **years** (1) ________________________________________________
- Prefer not to answer (-9)

| Page Break |  |
| --- | --- |

Display This Question:

If As of now, have you been abstinent from smoking cigars, cigarillos, or filtered cigars for at lea... = Yes

|  |  |  |  |
| --- | --- | --- | --- |

G08_d Think back to when you smoked cigars, cigarillos, or filtered cigars. On average, on those days you smoked, how many cigars, cigarillos, or filtered cigars did you usually smoke each day?

- Enter number of **cigars, cigarillos, or filtered cigars per day** (1) ________________________________________________
- Prefer not to answer (-9)

| Page Break |  |
| --- | --- |

Display This Question:

If In your lifetime, have you ever tried the following tobacco products, even just one time? Please... = Pipes

G_INTRO_PIPES **These questions will ask about your use of pipes and pipe tobacco.**

| Page Break |  |
| --- | --- |

Display This Question:

If In your lifetime, have you ever tried the following tobacco products, even just one time? Please... = Pipes

|  |  |
| --- | --- |

G09 Have you smoked 100 or more bowls filled with pipe tobacco in your **entire life**?

- Yes (1)
- No (0)
- Prefer not to answer (-9)

| Page Break |  |
| --- | --- |

Display This Question:

If In your lifetime, have you ever tried the following tobacco products, even just one time? Please... = Pipes

And Have you smoked 100 or more bowls filled with pipe tobacco in your entire life? = Yes

|  |  |
| --- | --- |

G10 Have you smoked a bowl filled with pipe tobacco in the**past 30 days**?

- Yes (1)
- No (0)
- Prefer not to answer (-9)

| Page Break |  |
| --- | --- |

Display This Question:

If Have you smoked a bowl filled with pipe tobacco in the past 30 days? = Yes

|  |  |  |  |
| --- | --- | --- | --- |

G10_b On how many of the **past 30 days** did you smoke a bowl filled with pipe tobacco?

- Enter number of **days** (1) ________________________________________________
- Prefer not to answer (-9)

| Page Break |  |
| --- | --- |

Display This Question:

If Have you smoked a bowl filled with pipe tobacco in the past 30 days? = Yes

|  |  |  |  |
| --- | --- | --- | --- |

G10_c On average, on those days you smoked, how many bowls filled with pipe tobacco did you usually smoke each day?

- Enter number of **bowls of pipe tobacco per day** (1) ________________________________________________
- Prefer not to answer (-9)

| Page Break |  |
| --- | --- |

Display This Question:

If Have you smoked a bowl filled with pipe tobacco in the past 30 days? = Yes

|  |  |  |  |
| --- | --- | --- | --- |

G10_d How many **years** have you smoked bowls filled with pipe tobacco? Please **do not** count any time you may have stayed off smoking pipe tobacco.
 *If you have smoked less than 1 year, please enter 0.*

- Number of **years** (1) ________________________________________________
- Prefer not to answer (-9)

| Page Break |  |
| --- | --- |

Display This Question:

If Have you smoked a bowl filled with pipe tobacco in the past 30 days? = No

|  |  |
| --- | --- |

G11 **As of now**, have you been abstinent from smoking pipe tobacco for at least 30 days, and intend to completely quit?

- Yes (1)
- No (0)
- Prefer not to answer (-9)

| Page Break |  |
| --- | --- |

Display This Question:

If As of now, have you been abstinent from smoking pipe tobacco for at least 30 days, and intend to... = Yes

|  |  |  |  |
| --- | --- | --- | --- |

G11_b About how long has it been since you **last smoked** a bowl filled with pipe tobacco? *If you quit smoking less than 1 year ago, please enter 0.*

- Enter number of **years** (1) ________________________________________________
- Prefer not to answer (-9)

| Page Break |  |
| --- | --- |

Display This Question:

If As of now, have you been abstinent from smoking pipe tobacco for at least 30 days, and intend to... = Yes

|  |  |  |  |
| --- | --- | --- | --- |

G11_c How many **years** did you smoke bowls filled with pipe tobacco? Please **do not** count any time you may have stayed off smoking pipe tobacco.
 *If you smoked less than 1 year, please enter 0.*

- Number of **years** (1) ________________________________________________
- Prefer not to answer (-9)

| Page Break |  |
| --- | --- |

Display This Question:

If As of now, have you been abstinent from smoking pipe tobacco for at least 30 days, and intend to... = Yes

|  |  |  |  |
| --- | --- | --- | --- |

G11_d Think back to when you smoked bowls filled with pipe tobacco. On average, on those days you smoked, how many bowls filled with pipe tobacco did you usually smoke each day?

- Enter number of **bowls of pipe tobacco per day** (1) ________________________________________________
- Prefer not to answer (-9)

| Page Break |  |
| --- | --- |

Display This Question:

If In your lifetime, have you ever tried the following tobacco products, even just one time? Please... = Hookah

G_INTRO_HOOKAH **These questions will ask about your hookah use.**

| Page Break |  |
| --- | --- |

Display This Question:

If In your lifetime, have you ever tried the following tobacco products, even just one time? Please... = Hookah

|  |  |
| --- | --- |

G12 Have you smoked tobacco in a hookah 100 or more times in your **entire life**? Count each sitting or session where you smoked tobacco in a hookah, whether alone or with others.

- Yes (1)
- No (0)
- Prefer not to answer (-9)

| Page Break |  |
| --- | --- |

Display This Question:

If In your lifetime, have you ever tried the following tobacco products, even just one time? Please... = Hookah

And Have you smoked tobacco in a hookah 100 or more times in your entire life? Count each sitting or... = Yes

|  |  |
| --- | --- |

G13 Have you smoked tobacco in a hookah in the**past 30 days**?

- Yes (1)
- No (0)
- Prefer not to answer (-9)

| Page Break |  |
| --- | --- |

Display This Question:

If Have you smoked tobacco in a hookah in the past 30 days? = Yes

|  |  |  |  |
| --- | --- | --- | --- |

G13_b On how many of the past 30 days did you smoke tobacco in a hookah?

- Number of **days** (1) ________________________________________________
- Prefer not to answer (-9)

| Page Break |  |
| --- | --- |

Display This Question:

If Have you smoked tobacco in a hookah in the past 30 days? = Yes

|  |  |  |  |
| --- | --- | --- | --- |

G13_c On average, on those days you smoked, how many times was the hookah refilled with tobacco during a session?

- Number of **refills per session** (1) ________________________________________________
- Prefer not to answer (-9)

| Page Break |  |
| --- | --- |

Display This Question:

If Have you smoked tobacco in a hookah in the past 30 days? = Yes

|  |  |  |  |
| --- | --- | --- | --- |

G13_d How many **years** have you smoked tobacco in a hookah? Please **do not** count any time you may have stayed off using a hookah.
 *If you have smoked less than 1 year, please enter 0.*

- Number of **years** (1) ________________________________________________
- Prefer not to answer (-9)

| Page Break |  |
| --- | --- |

Display This Question:

If Have you smoked tobacco in a hookah in the past 30 days? = No

|  |  |
| --- | --- |

G14 **As of now**, have you been abstinent from smoking tobacco in a hookah for at least 30 days, and intend to completely quit?

- Yes (1)
- No (0)
- Prefer not to answer (-9)

| Page Break |  |
| --- | --- |

Display This Question:

If As of now, have you been abstinent from smoking tobacco in a hookah for at least 30 days, and int... = Yes

|  |  |  |  |
| --- | --- | --- | --- |

G14_b About how long has it been since you **last smoked** tobacco in a hookah? *If you quit less than 1 year ago, please enter 0.*

- Number of **years** (1) ________________________________________________
- Prefer not to answer (-9)

| Page Break |  |
| --- | --- |

Display This Question:

If As of now, have you been abstinent from smoking tobacco in a hookah for at least 30 days, and int... = Yes

|  |  |  |  |
| --- | --- | --- | --- |

G14_c How many **years** did you smoke hookah filled with tobacco? Please **do not** count any time you may have stayed off using a hookah.
 *If you smoked less than 1 year, please enter 0.*

- Number of **years** (1) ________________________________________________
- Prefer not to answer (-9)

| Page Break |  |
| --- | --- |

Display This Question:

If As of now, have you been abstinent from smoking tobacco in a hookah for at least 30 days, and int... = Yes

|  |  |  |  |
| --- | --- | --- | --- |

G14_d Think back to when you smoked tobacco in a hookah. On average, how many sessions did you smoke tobacco in a hookah each day?

- Number of **hookah sessions per day** (1) ________________________________________________
- Prefer not to answer (-9)

| Page Break |  |
| --- | --- |

Display This Question:

If In your lifetime, have you ever tried the following tobacco products, even just one time? Please... = Smokeless tobacco (dip, spit, moist snuff, pouches, and chewing tobacco)

G_INTRO_SMOKLESS **These questions will ask about your smokeless tobacco use.**
 *Smokeless tobacco includes dip, spit, moist snuff, pouches, and chewing tobacco.*

| Page Break |  |
| --- | --- |

Display This Question:

If In your lifetime, have you ever tried the following tobacco products, even just one time? Please... = Smokeless tobacco (dip, spit, moist snuff, pouches, and chewing tobacco)

|  |  |
| --- | --- |

G15 Have you used smokeless tobacco 100 or more times in your **entire life?**

- Yes (1)
- No (0)
- Prefer not to answer (-9)

| Page Break |  |
| --- | --- |

Display This Question:

If In your lifetime, have you ever tried the following tobacco products, even just one time? Please... = Smokeless tobacco (dip, spit, moist snuff, pouches, and chewing tobacco)

And Have you used smokeless tobacco 100 or more times in your entire life? = Yes

|  |  |
| --- | --- |

G16 Have you used smokeless tobacco in the**past 30 days**?

- Yes (1)
- No (0)
- Prefer not to answer (-9)

| Page Break |  |
| --- | --- |

Display This Question:

If Have you used smokeless tobacco in the past 30 days? = Yes

|  |  |  |  |
| --- | --- | --- | --- |

G16_b On how many of the **past 30 days** did you use smokeless tobacco?

- Number of **days** (1) ________________________________________________
- Prefer not to answer (-9)

| Page Break |  |
| --- | --- |

Display This Question:

If Have you used smokeless tobacco in the past 30 days? = Yes

|  |  |  |  |
| --- | --- | --- | --- |

G16_c On average, on those days you used smokeless tobacco, how many times did you use smokeless tobacco each day?

- Number of **smokeless tobacco uses per day** (1) ________________________________________________
- Prefer not to answer (-9)

| Page Break |  |
| --- | --- |

Display This Question:

If Have you used smokeless tobacco in the past 30 days? = Yes

|  |  |  |  |
| --- | --- | --- | --- |

G16_d How many **years** have you used smokeless tobacco? Please **do not** count any time you may have stayed off using smokeless tobacco.
 *If you have used less than 1 year, please enter 0.*

- Number of **years** (1) ________________________________________________
- Prefer not to answer (-9)

| Page Break |  |
| --- | --- |

Display This Question:

If Have you used smokeless tobacco in the past 30 days? = No

|  |  |
| --- | --- |

G17 **As of now**, have you been abstinent from using smokeless tobacco for at least 30 days, and intend to completely quit?

- Yes (1)
- No (0)
- Prefer not to answer (-9)

| Page Break |  |
| --- | --- |

Display This Question:

If As of now, have you been abstinent from using smokeless tobacco for at least 30 days, and intend... = Yes

|  |  |  |  |
| --- | --- | --- | --- |

G17_b About how long has it been since you **last used** smokeless tobacco? *If you quit less than 1 year ago, please enter 0.*

- Number of **years** (1) ________________________________________________
- Prefer not to answer (-9)

| Page Break |  |
| --- | --- |

Display This Question:

If As of now, have you been abstinent from using smokeless tobacco for at least 30 days, and intend... = Yes

|  |  |  |  |
| --- | --- | --- | --- |

G17_c How many **years** did you use smokeless tobacco? Please **do not** count any time you may have stayed off using smokeless tobacco.
 *If you used less than 1 year, please enter 0.*

- Number of **years** (1) ________________________________________________
- Prefer not to answer (-9)

| Page Break |  |
| --- | --- |

Display This Question:

If As of now, have you been abstinent from using smokeless tobacco for at least 30 days, and intend... = Yes

|  |  |  |  |
| --- | --- | --- | --- |

G17_d Think back to when you used smokeless tobacco. On average, how many times did you use smokeless tobacco each day?

- Number of **smokeless tobacco uses per day** (1) ________________________________________________
- Prefer not to answer (-9)

| Page Break |  |
| --- | --- |

Display This Question:

If In your lifetime, have you ever tried the following tobacco products, even just one time? Please... = Snus

G_INTRO_SNUS **These questions will ask about your snus use.**

| Page Break |  |
| --- | --- |

Display This Question:

If In your lifetime, have you ever tried the following tobacco products, even just one time? Please... = Snus

|  |  |
| --- | --- |

G18 Have you used snus 100 or more times in your **entire life**?

- Yes (1)
- No (0)
- Prefer not to answer (-9)

| Page Break |  |
| --- | --- |

Display This Question:

If In your lifetime, have you ever tried the following tobacco products, even just one time? Please... = Snus

And Have you used snus 100 or more times in your entire life? = Yes

|  |  |
| --- | --- |

G19 Have you used snus in the**past 30 days**?

- Yes (1)
- No (0)
- Prefer not to answer (-9)

| Page Break |  |
| --- | --- |

Display This Question:

If Have you used snus in the past 30 days? = Yes

|  |  |  |  |
| --- | --- | --- | --- |

G19_b On how many of the **past 30 days** did you use snus?

- Number of **days** (1) ________________________________________________
- Prefer not to answer (-9)

| Page Break |  |
| --- | --- |

Display This Question:

If Have you used snus in the past 30 days? = Yes

|  |  |  |  |
| --- | --- | --- | --- |

G19_c On average, on those days you used snus, how many times did you use each day?

- Number of **snus uses per day** (1) ________________________________________________
- Prefer not to answer (-9)

| Page Break |  |
| --- | --- |

Display This Question:

If Have you used snus in the past 30 days? = Yes

|  |  |  |  |
| --- | --- | --- | --- |

G19_d How many **years** have you used snus? Please **do not** count any time you may have stayed off using snus.
 *If you have used less than 1 year, please enter 0.*

- Number of **years** (1) ________________________________________________
- Prefer not to answer (-9)

| Page Break |  |
| --- | --- |

Display This Question:

If Have you used snus in the past 30 days? = No

|  |  |
| --- | --- |

G20 **As of now,** have you been abstinent from using snus for at least 30 days, and intend to completely quit?

- Yes (1)
- No (0)
- Prefer not to answer (-9)

| Page Break |  |
| --- | --- |

Display This Question:

If As of now, have you been abstinent from using snus for at least 30 days, and intend to completely... = Yes

|  |  |  |  |
| --- | --- | --- | --- |

G20_b About how long has it been since you **last used** snus? *If you quit less than 1 year ago, please enter 0.*

- Number of **years** (1) ________________________________________________
- Prefer not to answer (-9)

| Page Break |  |
| --- | --- |

Display This Question:

If As of now, have you been abstinent from using snus for at least 30 days, and intend to completely... = Yes

|  |  |  |  |
| --- | --- | --- | --- |

G20_c How many **years** did you use snus? Please **do not** count any time you may have stayed off using snus.
 *If you used less than 1 year, please enter 0.*

- Number of **years** (1) ________________________________________________
- Prefer not to answer (-9)

| Page Break |  |
| --- | --- |

Display This Question:

If As of now, have you been abstinent from using snus for at least 30 days, and intend to completely... = Yes

|  |  |  |  |
| --- | --- | --- | --- |

G20_d Think back to when you used snus. On average, how many times did you use snus each day?

- Number of **snus uses per day** (1) ________________________________________________
- Prefer not to answer (-9)

| Page Break |  |
| --- | --- |

Display This Question:

If In your lifetime, have you ever tried the following tobacco products, even just one time? Please... = Electronic nicotine products (vapes, e-cigarettes, e-cigars, or similar)

G_INTRO_ECIG **These questions will ask about your use of electronic tobacco products.**
 *Some electronic nicotine products can be bought as one-time, disposable products, while others can be bought as re-usable kits with a cartridge or tank system. This includes e-cigarettes, e-cigars, e-pipes, e-hookahs, hookah pens, vape pens. Some common brands include Buse, Blu, Logic, MarkTen, JUUL, NJOY, eGo, and iTaste.*

| Page Break |  |
| --- | --- |

Display This Question:

If In your lifetime, have you ever tried the following tobacco products, even just one time? Please... = Electronic nicotine products (vapes, e-cigarettes, e-cigars, or similar)

|  |  |
| --- | --- |

G21 Have you ever used an electronic nicotine product as a way of cutting down on or quitting cigarette smoking?

- Yes (1)
- No (0)
- Prefer not to answer (-9)

| Page Break |  |
| --- | --- |

Display This Question:

If In your lifetime, have you ever tried the following tobacco products, even just one time? Please... = Electronic nicotine products (vapes, e-cigarettes, e-cigars, or similar)

|  |  |
| --- | --- |

G22 Have you used electronic nicotine products 100 or more times in your **entire life**?

- Yes (1)
- No (0)
- Prefer not to answer (-9)

| Page Break |  |
| --- | --- |

Display This Question:

If In your lifetime, have you ever tried the following tobacco products, even just one time? Please... = Electronic nicotine products (vapes, e-cigarettes, e-cigars, or similar)

And Have you used electronic nicotine products 100 or more times in your entire life? = Yes

|  |  |
| --- | --- |

G23 Have you used an electronic nicotine product in the**past 30 days**?

- Yes (1)
- No (0)
- Prefer not to answer (-9)

| Page Break |  |
| --- | --- |

Display This Question:

If Have you used an electronic nicotine product in the past 30 days? = Yes

|  |  |  |  |
| --- | --- | --- | --- |

G23_b On how many of the **past 30 days** did you use an electronic nicotine product?

- Number of **days** (1) ________________________________________________
- Prefer not to answer (-9)

| Page Break |  |
| --- | --- |

Display This Question:

If Have you used an electronic nicotine product in the past 30 days? = Yes

|  |  |  |  |
| --- | --- | --- | --- |

G23_c How many **years** have you used an electronic nicotine product? Please **do not** count any time you may have stayed off using an electronic nicotine product.
 *If you have used less than 1 year, please enter 0.*

- Number of **years** (1) ________________________________________________
- Prefer not to answer (-9)

| Page Break |  |
| --- | --- |

Display This Question:

If Have you used an electronic nicotine product in the past 30 days? = Yes

|  |  |  |  |
| --- | --- | --- | --- |

G23_d On average, on those days you picked up an electronic nicotine product, about how many puffs did you take each time?

- Number of **puffs per use** (1) ________________________________________________
- Prefer not to answer (-9)

| Page Break |  |
| --- | --- |

Display This Question:

If Have you used an electronic nicotine product in the past 30 days? = No

|  |  |
| --- | --- |

G24 **As of now**, have you been abstinent from using electronic nicotine products for at least 30 days, and intend to completely quit?

- Yes (1)
- No (0)
- Prefer not to answer (-9)

| Page Break |  |
| --- | --- |

Display This Question:

If As of now, have you been abstinent from using electronic nicotine products for at least 30 days,... = Yes

|  |  |  |  |
| --- | --- | --- | --- |

G24_b How many **years** did you use an electronic nicotine product? Please **do not** count any time you may have stayed off using an electronic nicotine product.
 *If you have used less than 1 year, please enter 0.*

- Number of **years** (1) ________________________________________________
- Prefer not to answer (-9)

| Page Break |  |
| --- | --- |

Display This Question:

If As of now, have you been abstinent from using electronic nicotine products for at least 30 days,... = Yes

|  |  |  |  |
| --- | --- | --- | --- |

G24_c About how long has it been since you **last used** an electronic nicotine product? *If you quit less than 1 year ago, please enter 0.*

- Number of **years** (1) ________________________________________________
- Prefer not to answer (-9)

| Page Break |  |
| --- | --- |

Display This Question:

If As of now, have you been abstinent from using electronic nicotine products for at least 30 days,... = Yes

|  |  |  |  |
| --- | --- | --- | --- |

G24_d Think back to when you used an electronic nicotine products. On average, how many puffs times did you take each time?

- Number of **puffs per use** (1) ________________________________________________
- Prefer not to answer (-9)

| Page Break |  |
| --- | --- |

Display This Question:

If Have you used electronic nicotine products 100 or more times in your entire life? = Yes

|  |  |  |
| --- | --- | --- |

G25 Typically, when you use(d) electronic nicotine products, which flavor(s) do/did you use?
 *(Check all that apply)*

- Tobacco flavored (0)
- Menthol or mint (1)
- Clove or spice (2)
- Fruit (3)
- Chocolate (4)
- An alcoholic drink (such as wine, cognac, margarita, or other cocktails) (5)
- A non-alcoholic drink (such as coffee, soda, energy drinks, or other beverages) (6)
- Candy, desserts, or other sweets (7)
- Not listed (please specify): (-8) ________________________________________________
- ⊗Prefer not to answer (-9)

| Page Break |  |
| --- | --- |

Display This Question:

If Have you used electronic nicotine products 100 or more times in your entire life? = Yes

|  |  |
| --- | --- |

G26 How often do/did you refill your electronic nicotine product with e-liquid?

- At least once a day (0)
- Once every couple of days (1)
- Once a week (2)
- A few times a month (3)
- Once a month (4)
- A few times a year (5)
- Once a year (6)
- Prefer not to answer (-9)

| Page Break |  |
| --- | --- |

Display This Question:

If Have you used electronic nicotine products 100 or more times in your entire life? = Yes

|  |  |  |
| --- | --- | --- |

G27 Where do/did you buy your e-liquid most of the time?

- A vape shop or vapor lounge (0)
- A mall kiosk (1)
- A convenience store or gas station (2)
- A supermarket, grocery store, or drug store (3)
- A warehouse club, such as Sam's or Costco (4)
- A smoke shop, tobacco specialty store, or tobacco outlet store (5)
- A duty-free shop or military commissary (6)
- A bar, pub, restaurant, or casino (7)
- A friend or relative (8)
- A swap meet or flea market (9)
- A liquor store (10)
- Not listed (please specify): (-8) ________________________________________________
- Prefer not to answer (-9)

| Page Break |  |
| --- | --- |

Display This Question:

If If In your lifetime, have you ever tried the following tobacco products, even just one time? Please... Text Response Is Not Empty

G_INTRO_TEXT **These questions will ask about your use of ${G01/ChoiceTextEntryValue/8}.**

| Page Break |  |
| --- | --- |

Display This Question:

If If In your lifetime, have you ever tried the following tobacco products, even just one time? Please... Text Response Is Not Empty

|  |  |
| --- | --- |

G28 Have you used ${G01/ChoiceTextEntryValue/8} 100 or more times in your **entire life**?

- Yes (1)
- No (0)
- Prefer not to answer (-9)

| Page Break |  |
| --- | --- |

Display This Question:

If If In your lifetime, have you ever tried the following tobacco products, even just one time? Please... Text Response Is Not Empty

And Have you used ${q://QID101/ChoiceTextEntryValue/8} 100 or more times in your entire life? = Yes

|  |  |
| --- | --- |

G29 Have you used ${G01/ChoiceTextEntryValue/8} in the **past 30 days**?

- Yes (1)
- No (0)
- Prefer not to answer (-9)

| Page Break |  |
| --- | --- |

Display This Question:

If Have you used ${q://QID101/ChoiceTextEntryValue/8} in the past 30 days? = Yes

|  |  |  |  |
| --- | --- | --- | --- |

G29_b On how many of the **past 30 days** have you used ${G01/ChoiceTextEntryValue/8}?

- Number of **days** (1) ________________________________________________
- Prefer not to answer (-9)

| Page Break |  |
| --- | --- |

Display This Question:

If Have you used ${q://QID101/ChoiceTextEntryValue/8} in the past 30 days? = Yes

|  |  |  |  |
| --- | --- | --- | --- |

G29_c On average, on those days you used ${G01/ChoiceTextEntryValue/8}, how many times did you use ${G01/ChoiceTextEntryValue/8} per day?

- Number of **uses per day** (1) ________________________________________________
- Prefer not to answer (-9)

| Page Break |  |
| --- | --- |

Display This Question:

If Have you used ${q://QID101/ChoiceTextEntryValue/8} in the past 30 days? = Yes

|  |  |  |  |
| --- | --- | --- | --- |

G29_d How many **years** have you used ${G01/ChoiceTextEntryValue/8}? Please do not count any time you may have stayed off ${G01/ChoiceTextEntryValue/8}. *If you have used less than 1 year, please enter 0.*

- Number of **years** (1) ________________________________________________
- Prefer not to answer (-9)

| Page Break |  |
| --- | --- |

Display This Question:

If Have you used ${q://QID101/ChoiceTextEntryValue/8} in the past 30 days? = No

|  |  |
| --- | --- |

G30 **As of now**, have you been abstinent from using ${G01/ChoiceTextEntryValue/8} for at least 30 days, and intend to completely quit?

- Yes (1)
- No (0)
- Prefer not to answer (-9)

| Page Break |  |
| --- | --- |

Display This Question:

If As of now, have you been abstinent from using ${q://QID101/ChoiceTextEntryValue/8} for at least 3... = Yes

|  |  |  |  |
| --- | --- | --- | --- |

G30_b About how long has it been since you **last used** ${G01/ChoiceTextEntryValue/8}? *If you quit less than 1 year ago, please enter 0.*

- Number of **years** (1) ________________________________________________
- Prefer not to answer (-9)

| Page Break |  |
| --- | --- |

Display This Question:

If As of now, have you been abstinent from using ${q://QID101/ChoiceTextEntryValue/8} for at least 3... = Yes

|  |  |  |  |
| --- | --- | --- | --- |

G30_c How many **years** did you use ${G01/ChoiceTextEntryValue/8}? Please do not count any time you may have stayed off ${G01/ChoiceTextEntryValue/8}. *If you used less than 1 year, please enter 0.*

- Number of **years** (1) ________________________________________________
- Prefer not to answer (-9)

| Page Break |  |
| --- | --- |

Display This Question:

If As of now, have you been abstinent from using ${q://QID101/ChoiceTextEntryValue/8} for at least 3... = Yes

|  |  |  |  |
| --- | --- | --- | --- |

G30_d Think back to when you used ${G01/ChoiceTextEntryValue/8}. On average, how many times did you use ${G01/ChoiceTextEntryValue/8} each day?

- Number of **uses per day** (1) ________________________________________________
- Prefer not to answer (-9)

End of Block: G_Tobacco

Start of Block: H_Alcohol

H_INTRO **You are making great progress!This section will ask about your previous and current consumption of alcohol.**You are over halfway through the survey!

| Page Break |  |
| --- | --- |

|  |  |
| --- | --- |

H01 **In the past year,** how often did you have a drink containing alcohol?

- I have never drank alcohol in my life (0)
- I did not drink alcohol in the past year, but I did drink in the past (1)
- Monthly or less (2)
- 2 to 4 times a month (3)
- 2 to 3 times a week (4)
- 4 or more times a week (5)
- Prefer not to answer (-9)

Skip To: End of Block If In the past year, how often did you have a drink containing alcohol? = I have never drank alcohol in my life

Skip To: End of Block If In the past year, how often did you have a drink containing alcohol? = Prefer not to answer

| Page Break |  |
| --- | --- |

Display This Question:

If In the past year, how often did you have a drink containing alcohol? = Monthly or less

Or In the past year, how often did you have a drink containing alcohol? = 2 to 4 times a month

Or In the past year, how often did you have a drink containing alcohol? = 2 to 3 times a week

Or In the past year, how often did you have a drink containing alcohol? = 4 or more times a week

|  |  |  |  |
| --- | --- | --- | --- |

H02 **In the past year**, how many standard drinks containing alcohol did you have on a **typical day** when you were drinking?

- Number of standard drinks (1) ________________________________________________
- Prefer not to answer (-9)

| Page Break |  |
| --- | --- |

Display This Question:

If If In the past year, how many standard drinks containing alcohol did you have on a typical day when... Text Response Is Not Empty

|  |  |
| --- | --- |

H02_b **In the past year**, did you have ${H02/ChoiceTextEntryValue/1} drinks almost every day?

- Yes (1)
- No (0)
- Prefer not to answer (-9)

| Page Break |  |
| --- | --- |

Display This Question:

If In the past year, how often did you have a drink containing alcohol? = Monthly or less

Or In the past year, how often did you have a drink containing alcohol? = 2 to 4 times a month

Or In the past year, how often did you have a drink containing alcohol? = 2 to 3 times a week

Or In the past year, how often did you have a drink containing alcohol? = 4 or more times a week

|  |  |
| --- | --- |

H03 **In the past year**, how often did you have **6 or more standard drinks** on one occasion?

- Never (0)
- Less than monthly (1)
- Monthly or less (2)
- Weekly (3)
- Daily or almost daily (4)
- Always (5)
- Prefer not to answer (-9)

| Page Break |  |
| --- | --- |

Display This Question:

If In the past year, how often did you have a drink containing alcohol? = I did not drink alcohol in the past year, but I did drink in the past

|  |  |  |  |
| --- | --- | --- | --- |

H04 How many **years** has it been since you last had a beverage containing alcohol?

- Number of **years** (1) ________________________________________________
- Prefer not to answer (-9)

| Page Break |  |
| --- | --- |

Display This Question:

If In the past year, how often did you have a drink containing alcohol? = I did not drink alcohol in the past year, but I did drink in the past

Or In the past year, how often did you have a drink containing alcohol? = Monthly or less

Or In the past year, how often did you have a drink containing alcohol? = 2 to 4 times a month

Or In the past year, how often did you have a drink containing alcohol? = 2 to 3 times a week

Or In the past year, how often did you have a drink containing alcohol? = 4 or more times a week

|  |  |
| --- | --- |

H03_b **In your life,** has there ever been a period where you drank **6 or more standard drinks** of any alcoholic beverage **almost every day**?

- Yes (1)
- No (0)
- Prefer not to answer (-9)

| Page Break |  |
| --- | --- |

Display This Question:

If In your life, has there ever been a period where you drank 6 or more standard drinks of any alcoh... = Yes

|  |  |  |  |
| --- | --- | --- | --- |

H05_c During this period of your life, on the days you had a beverage containing alcohol, about **how many standard drinks** did you have on average?

- Number of **drinks per day** (1) ________________________________________________
- Prefer not to answer (-9)

Display This Question:

If In your life, has there ever been a period where you drank 6 or more standard drinks of any alcoh... = Yes

|  |  |  |  |
| --- | --- | --- | --- |

H05_d How many **years** did this period last?  *If less than 1 year, please enter 0.*

- Number of **years** (1) ________________________________________________
- Prefer not to answer (-9)

| Page Break |  |
| --- | --- |

Display This Question:

If In the past year, how often did you have a drink containing alcohol? = I did not drink alcohol in the past year, but I did drink in the past

Or In the past year, how often did you have a drink containing alcohol? = Monthly or less

Or In the past year, how often did you have a drink containing alcohol? = 2 to 4 times a month

Or In the past year, how often did you have a drink containing alcohol? = 2 to 3 times a week

Or In the past year, how often did you have a drink containing alcohol? = 4 or more times a week

|  |  |
| --- | --- |

H05 **In your life**, have you ever drunk as much as a fifth of liquor in one day (about 20 drinks), or 3 bottles of wine, or as much as 3 six-packs of beer **in a single day**?

- Yes (1)
- No (0)
- Prefer not to answer (-9)

Skip To: End of Block If In your life, have you ever drunk as much as a fifth of liquor in one day (about 20 drinks), or 3... = Prefer not to answer

| Page Break |  |
| --- | --- |

Display This Question:

If In your life, have you ever drunk as much as a fifth of liquor in one day (about 20 drinks), or 3... = Yes

|  |  |
| --- | --- |

H05_b **How** **often** did you drink a fifth of liquor, or 3 bottles of wine, or as much as 3 six-packs of beer **in a single day**?

- Monthly or less (0)
- 2 to 4 times a month (1)
- 2 to 3 times a week (2)
- 4 or more times a week (3)
- Prefer not to answer (-9)

Display This Question:

If In your life, have you ever drunk as much as a fifth of liquor in one day (about 20 drinks), or 3... = Yes

|  |  |  |  |
| --- | --- | --- | --- |

H05_c How many **years** did this period last? *If less than 1 year, please enter 0.*

- Number of **years** (1) ________________________________________________
- Prefer not to answer (-9)

End of Block: H_Alcohol

Start of Block: I_Sexual Behaviors

I_INTRO **This section will ask about your sexual partners and behaviors. All data is confidential and we greatly appreciate your honest responses.**
  Remember, you can save and return to this section if you need a break.

| Page Break |  |
| --- | --- |

|  |  |
| --- | --- |

I01 When was the last time you had any type of sex with a man?

- Within the past 12 months (0)
- Between 1 to 5 years ago (1)
- More than 5 years ago (2)

| Page Break |  |
| --- | --- |

|  |  |
| --- | --- |

I02 **In your life**, with how many **different cisgender men** have you had any kind of sex (including masturbating each other, oral, or anal sex)?

 *Cisgender man: the partner was assigned male at birth and identifies as a man*

- None, I have not had sex with a man (0)
- 1-10 men (1)
- 11-20 men (2)
- 21-100 men (3)
- 101-1,000 men (4)
- 1,001-10,000 men (5)
- More than 10,000 men (6)
- Prefer not to answer (-9)

| Page Break |  |
| --- | --- |

Display This Question:

If In your life, with how many different cisgender men have you had any kind of sex (including mastu... = 1-10 men

Or In your life, with how many different cisgender men have you had any kind of sex (including mastu... = 11-20 men

Or In your life, with how many different cisgender men have you had any kind of sex (including mastu... = 21-100 men

Or In your life, with how many different cisgender men have you had any kind of sex (including mastu... = 101-1,000 men

Or In your life, with how many different cisgender men have you had any kind of sex (including mastu... = 1,001-10,000 men

Or In your life, with how many different cisgender men have you had any kind of sex (including mastu... = More than 10,000 men

Or In your life, with how many different cisgender men have you had any kind of sex (including mastu... = Prefer not to answer

|  |  |  |  |
| --- | --- | --- | --- |

I02_b **In the last 12 months**, with how many **different cisgender men** have you had any kind of sex (including masturbating each other, oral, or anal sex)?

 *Cisgender man: the partner was assigned male at birth and identifies as a man*

- Number of men in last 12 months (1) ________________________________________________
- None, I have not had sex with a man in the last 12 months. (0)
- Prefer not to answer (-9)

| Page Break |  |
| --- | --- |

|  |  |  |  |
| --- | --- | --- | --- |

I03 **In your life**, with how many **different cisgender women** have you had any kind of sex (including jerking off/fingering each other, oral, vaginal, or anal sex)?

 *Cisgender woman: the partner was assigned female at birth and identifies as a woman*

- Number of women (1) ________________________________________________
- None, I have not had sex with a woman. (0)
- Prefer not to answer (-9)

| Page Break |  |
| --- | --- |

Display This Question:

If If In your life, with how many different cisgender women have you had any kind of sex (including jer... Text Response Is Greater Than 0

|  |  |  |  |
| --- | --- | --- | --- |

I03_b **In the last 12 months**, with how many **different cisgender women** have you had any kind of sex (including jerking off/fingering each other, oral, vaginal, or anal sex)?

 *Cisgender woman: the partner was assigned female at birth and identifies as a woman*

- Number of women in last 12 months (1) ________________________________________________
- None, I have not had sex with a woman in the last 12 months. (0)
- Prefer not to answer (-9)

| Page Break |  |
| --- | --- |

|  |  |  |  |
| --- | --- | --- | --- |

I04 **In your life**, with how many **different transgender or non-binary persons** have you had any kind of sex (including jerking off/fingering each other, oral, vaginal, or anal sex)?

 *Transgender or gender non-binary: the partner’s sex assigned at birth does not align with their current gender identity (i.e. transgender men, transgender women, gender non-conforming…)*

- Number of transgender or non-binary persons (1) ________________________________________________
- None, I have not had sex with a transgender or non-binary person. (0)
- Prefer not to answer (-9)

| Page Break |  |
| --- | --- |

Display This Question:

If If In your life, with how many different transgender or non-binary persons have you had any kind of... Text Response Is Greater Than 0

|  |  |  |  |
| --- | --- | --- | --- |

I04_b **In the last 12 months**, with how many **different transgender or non-binary persons** have you had any kind of sex (including jerking off/fingering each other, oral, vaginal, or anal sex)?

 *Transgender or gender non-binary: the partner’s sex assigned at birth does not align with their current gender identity (i.e. transgender men, transgender women, gender non-conforming…)*

- Number of transgender or non-binary persons in last 12 months (1) ________________________________________________
- None, I have not had sex with a transgender or non-binary person in the last 12 months. (0)
- Prefer not to answer (-9)

| Page Break |  |
| --- | --- |

I_INTRO_BJS The following questions will ask about your experiences **giving blowjobs** or performing oral sex on someone with a penis.

| Page Break |  |
| --- | --- |

|  |  |
| --- | --- |

I05 **In your lifetime**, with how many different partners have **you given blowjobs or performed oral sex on** (your mouth on their penis)?
 *This can be blowjobs with or without orgasm.*

- None (0)
- 1-10 partners (1)
- 11-20 partners (2)
- 21-100 partners (3)
- 101-1,000 partners (4)
- 1,001-10,000 partners (5)
- More than 10,000 partners (6)
- Prefer not to answer (-9)

Skip To: I_INTRO_RIMMING If In your lifetime, with how many different partners have you given blowjobs or performed oral sex... = None

| Page Break |  |
| --- | --- |

Display This Question:

If In your lifetime, with how many different partners have you given blowjobs or performed oral sex... != None

|  |  |  |  |
| --- | --- | --- | --- |

I05_b In the **last 12 months**, with how many different partners have **you given blowjobs or performed oral sex on**(your mouth on their penis)?
 *This can be blowjobs with or without orgasm.*

- Number of partners (1) ________________________________________________
- None, I have not given blowjobs in the last 12 months. (0)
- Prefer not to answer (-9)

Skip To: I_INTRO_RIMMING If In the last 12 months, with how many different partners have you given blowjobs or performed oral... = None, I have not given blowjobs in the last 12 months.

Skip To: I_INTRO_RIMMING If In the last 12 months, with how many different partners have you given blowjobs or performed oral... = Prefer not to answer

| Page Break |  |
| --- | --- |

Display This Question:

If If In the last 12 months, with how many different partners have you given blowjobs or performed oral... Text Response Is Greater Than or Equal to 1

|  |  |
| --- | --- |

I06 In the **last 12 months**, how frequently did **you give blowjobs** or perform oral sex on your partner(s) (your mouth on their penis)?
 *This can be blowjobs with or without orgasm.*

- Daily to several times a day (0)
- Several times a week (1)
- About once a week (2)
- About once a month (3)
- At least once a year (4)
- Prefer not to answer (-9)

| Page Break |  |
| --- | --- |

Display This Question:

If If In the last 12 months, with how many different partners have you given blowjobs or performed oral... Text Response Is Greater Than or Equal to 1

|  |  |
| --- | --- |

I07 In the **last 12 months**, how important was giving blowjobs or performing oral sex on your partner(s) (your mouth on their penis) to you?
 *This can be blowjobs with or without orgasm.*

- Not important at all (I don’t need it) (0)
- Slightly important (it’s nice for a change) (1)
- Moderately important (I like doing it regularly) (2)
- Important (I really prefer it) (3)
- Very important (it’s central to my sex life) (4)
- Prefer not to answer (-9)

| Page Break |  |
| --- | --- |

Display This Question:

If If In the last 12 months, with how many different partners have you given blowjobs or performed oral... Text Response Is Greater Than or Equal to 1

|  |  |
| --- | --- |

I08 In the **last 12 months**, how often did you check your mouth for sores or cuts before or after giving blowjobs?
 *This can be blowjobs with or without orgasm.*

- Never (or almost never) (0)
- Seldom (less than 25% of occasions) (1)
- Sometimes (26-50% of occasions) (2)
- Often (51-75% of occasions) (3)
- Usually (76-99% of occasions) (4)
- Always (or almost always) (5)
- Prefer not to answer (-9)

| Page Break |  |
| --- | --- |

Display This Question:

If In the last 12 months, how often did you check your mouth for sores or cuts before or after givin... = Seldom (less than 25% of occasions)

Or In the last 12 months, how often did you check your mouth for sores or cuts before or after givin... = Sometimes (26-50% of occasions)

Or In the last 12 months, how often did you check your mouth for sores or cuts before or after givin... = Often (51-75% of occasions)

Or In the last 12 months, how often did you check your mouth for sores or cuts before or after givin... = Usually (76-99% of occasions)

Or In the last 12 months, how often did you check your mouth for sores or cuts before or after givin... = Always (or almost always)

|  |  |  |
| --- | --- | --- |

I08_b Please tell us all the ways you checked your mouth before or after giving blowjobs or oral sex. *This can be blowjobs with or without orgasm.*
 *(Check all that apply)*

- Wash my mouth with mouthwash (0)
- Brush my teeth with toothpaste (1)
- Visually check my mouth in a mirror (2)
- Run my tongue around my mouth to feel for bumps and cuts (3)
- Take a photo ("selfie") of my mouth and examine the picture (4)
- Taste for blood (5)
- Not listed (please specify): (-8) ________________________________________________
- ⊗Prefer not to answer (-9)

| Page Break |  |
| --- | --- |

Display This Question:

If If In the last 12 months, with how many different partners have you given blowjobs or performed oral... Text Response Is Greater Than or Equal to 1

|  |  |
| --- | --- |

I09 In the **last 12 months**, when you gave blowjobs or oral sex, how often did the penis rub the back of your throat?
 *This can be blowjobs with or without orgasm.*

- Never (or almost never) (0)
- Seldom (less than 25% of occasions) (1)
- Sometimes (26-50% of occasions) (2)
- Often (51-75% of occasions) (3)
- Usually (76-99% of occasions) (4)
- Always (or almost always) (5)
- Prefer not to answer (-9)

| Page Break |  |
| --- | --- |

Display This Question:

If If In the last 12 months, with how many different partners have you given blowjobs or performed oral... Text Response Is Greater Than or Equal to 1

|  |  |
| --- | --- |

I10 In the **last 12 months**, when you gave blowjobs or oral sex, how often did you deep throat the penis?
 *This can be blowjobs with or without orgasm.*

- Never (or almost never) (0)
- Seldom (less than 25% of occasions) (1)
- Sometimes (26-50% of occasions) (2)
- Often (51-75% of occasions) (3)
- Usually (76-99% of occasions) (4)
- Always (or almost always) (5)
- Prefer not to answer (-9)

| Page Break |  |
| --- | --- |

Display This Question:

If If In the last 12 months, with how many different partners have you given blowjobs or performed oral... Text Response Is Greater Than or Equal to 1

|  |  |
| --- | --- |

I11 In the **last 12 months**, when you gave blowjobs or oral sex, how often did you have a sore throat afterwards?
 *This can be blowjobs with or without orgasm.*

- Never (or almost never) (0)
- Seldom (less than 25% of occasions) (1)
- Sometimes (26-50% of occasions) (2)
- Often (51-75% of occasions) (3)
- Usually (76-99% of occasions) (4)
- Always (or almost always) (5)
- Prefer not to answer (-9)

| Page Break |  |
| --- | --- |

Display This Question:

If If In the last 12 months, with how many different partners have you given blowjobs or performed oral... Text Response Is Greater Than or Equal to 1

|  |  |
| --- | --- |

I12 In the **last 12 months**, when you gave blowjobs or oral sex, how often was the penis been uncircumcised?
 *This can be blowjobs with or without orgasm.*

- Never (or almost never) (0)
- Seldom (less than 25% of occasions) (1)
- Sometimes (26-50% of occasions) (2)
- Often (51-75% of occasions) (3)
- Usually (76-99% of occasions) (4)
- Always (or almost always) (5)
- Prefer not to answer (-9)

| Page Break |  |
| --- | --- |

Display This Question:

If If In the last 12 months, with how many different partners have you given blowjobs or performed oral... Text Response Is Greater Than or Equal to 1

|  |  |
| --- | --- |

I13 In the **last 12 months**, when you gave blowjobs or oral sex, how often did you swallow the ejaculate (cum)?

- Never (or almost never) (0)
- Seldom (less than 25% of occasions) (1)
- Sometimes (26-50% of occasions) (2)
- Often (51-75% of occasions) (3)
- Usually (76-99% of occasions) (4)
- Always (or almost always) (5)
- Prefer not to answer (-9)

| Page Break |  |
| --- | --- |

Display This Question:

If If In the last 12 months, with how many different partners have you given blowjobs or performed oral... Text Response Is Greater Than or Equal to 1

|  |  |
| --- | --- |

I14 In the **last 12 months**, how often did you worry about possibly getting a sexually transmitted infection (STI) from giving blowjobs to your partner(s)?
 *This can be blowjobs with or without orgasm.*

- Never (or almost never) (0)
- Seldom (less than 25% of occasions) (1)
- Sometimes (26-50% of occasions) (2)
- Often (51-75% of occasions) (3)
- Usually (76-99% of occasions) (4)
- Always (or almost always) (5)
- Prefer not to answer (-9)

| Page Break |  |
| --- | --- |

I_INTRO_RIMMING The following questions will ask about your experiences **rimming** or performing oral sex on a partner's anus.

| Page Break |  |
| --- | --- |

|  |  |
| --- | --- |

I15 **In your lifetime**, how many different partners **have you rimmed** or performed oral sex on their anus (your mouth on their anus)?

- None (0)
- 1-10 partners (1)
- 11-20 partners (2)
- 21-100 partners (3)
- 101-1,000 partners (4)
- 1,001-10,000 partners (5)
- More than 10,000 partners (6)
- Prefer not to answer (-9)

Skip To: I_INTRO_EATOUT If In your lifetime, how many different partners have you rimmed or performed oral sex on their anus... = None

| Page Break |  |
| --- | --- |

Display This Question:

If In your lifetime, how many different partners have you rimmed or performed oral sex on their anus... != None

|  |  |  |  |
| --- | --- | --- | --- |

I15_b In the **last 12 months**, how many different partners have **you rimmed or performed oral sex on** (your mouth on their anus)?

- Number of partners (1) ________________________________________________
- None, I have not rimmed a partner in the last 12 months. (0)
- Prefer not to answer (-9)

Skip To: I_INTRO_EATOUT If In the last 12 months, how many different partners have you rimmed or performed oral sex on (your... = None, I have not rimmed a partner in the last 12 months.

Skip To: I_INTRO_EATOUT If In the last 12 months, how many different partners have you rimmed or performed oral sex on (your... = Prefer not to answer

| Page Break |  |
| --- | --- |

Display This Question:

If If In the last 12 months, how many different partners have you rimmed or performed oral sex on (your... Text Response Is Greater Than 0

|  |  |
| --- | --- |

I16 In the **last 12 months**, how frequently did **you rim** your partner(s) or perform oral sex on their anus?

- Daily to several times a day (0)
- Several times a week (1)
- About once a week (2)
- About once a month (3)
- At least once a year (4)
- Prefer not to answer (-9)

| Page Break |  |
| --- | --- |

Display This Question:

If If In the last 12 months, how many different partners have you rimmed or performed oral sex on (your... Text Response Is Greater Than 0

|  |  |
| --- | --- |

I17 In the **last 12 months**, how important was rimming your partner(s) (your mouth on their anus) to you?

- Not important at all (I don’t need it) (0)
- Slightly important (it’s nice for a change) (1)
- Moderately important (I like doing it regularly) (2)
- Important (I really prefer it) (3)
- Very important (it’s central to my sex life) (4)
- Prefer not to answer (-9)

| Page Break |  |
| --- | --- |

Display This Question:

If If In the last 12 months, how many different partners have you rimmed or performed oral sex on (your... Text Response Is Greater Than 0

|  |  |
| --- | --- |

I18 In the **last 12 months**, how often did you check your mouth for sores or cuts before or after rimming your partner(s)?

- Never (or almost never) (0)
- Seldom (less than 25% of occasions) (1)
- Sometimes (26-50% of occasions) (2)
- Often (51-75% of occasions) (3)
- Usually (76-99% of occasions) (4)
- Always (or almost always) (5)
- Prefer not to answer (-9)

| Page Break |  |
| --- | --- |

Display This Question:

If In the last 12 months, how often did you check your mouth for sores or cuts before or after rimmi... = Seldom (less than 25% of occasions)

Or In the last 12 months, how often did you check your mouth for sores or cuts before or after rimmi... = Sometimes (26-50% of occasions)

Or In the last 12 months, how often did you check your mouth for sores or cuts before or after rimmi... = Often (51-75% of occasions)

Or In the last 12 months, how often did you check your mouth for sores or cuts before or after rimmi... = Usually (76-99% of occasions)

Or In the last 12 months, how often did you check your mouth for sores or cuts before or after rimmi... = Always (or almost always)

|  |  |  |
| --- | --- | --- |

I18_b Please tell us all the ways you checked your mouth after rimming your partner(s).
 *(Check all that apply)*

- Wash my mouth with mouthwash (0)
- Brush my teeth with toothpaste (1)
- Visually check my mouth in a mirror (2)
- Run my tongue around my mouth to feel for bumps and cuts (3)
- Take a photo ("selfie") of my mouth and examine the picture (4)
- Taste for blood (5)
- Not listed (please specify): (-8) ________________________________________________
- ⊗Prefer not to answer (-9)

| Page Break |  |
| --- | --- |

Display This Question:

If If In the last 12 months, how many different partners have you rimmed or performed oral sex on (your... Text Response Is Greater Than 0

|  |  |
| --- | --- |

I19 In the **last 12 months**, how often did you worry about possibly getting a sexually transmitted infection (STI) from rimming your partner(s)?

- Never (or almost never) (0)
- Seldom (less than 25% of occasions) (1)
- Sometimes (26-50% of occasions) (2)
- Often (51-75% of occasions) (3)
- Usually (76-99% of occasions) (4)
- Always (or almost always) (5)
- Prefer not to answer (-9)

| Page Break |  |
| --- | --- |

I_INTRO_EATOUT The following questions will ask about your experiences **eating out your partner(s)** or performing oral sex on someone with a vagina.

| Page Break |  |
| --- | --- |

|  |  |
| --- | --- |

I20 **In your lifetime**, how many different partners **have you eaten out** or performed oral sex on their vagina (your mouth on their vagina)?*This can be with or without orgasm.*

- None (0)
- 1-10 partners (1)
- 11-20 partners (2)
- 21-100 partners (3)
- 101-1,000 partners (4)
- 1,001-10,000 partners (5)
- More than 10,000 partners (6)
- Prefer not to answer (-9)

Skip To: I_INTRO_RECIPIENT If In your lifetime, how many different partners have you eaten out or performed oral sex on their v... = None

| Page Break |  |
| --- | --- |

Display This Question:

If In your lifetime, how many different partners have you eaten out or performed oral sex on their v... != None

|  |  |  |  |
| --- | --- | --- | --- |

I20_b In the **last 12 months**, how many different partners **have you eaten out** or performed oral sex on (your mouth on their vagina)?*This can be with or without orgasm.*

- Number of partners (1) ________________________________________________
- None, I have not eaten out a partner in the last 12 months. (0)
- Prefer not to answer (-9)

Skip To: I_INTRO_RECIPIENT If In the last 12 months, how many different partners have you eaten out or performed oral sex on (y... = None, I have not eaten out a partner in the last 12 months.

Skip To: I_INTRO_RECIPIENT If In the last 12 months, how many different partners have you eaten out or performed oral sex on (y... = Prefer not to answer

| Page Break |  |
| --- | --- |

Display This Question:

If If In the last 12 months, how many different partners have you eaten out or performed oral sex on (y... Text Response Is Greater Than 0

|  |  |
| --- | --- |

I21 In the **last 12 months**, how frequently did y**ou eat out** or perform oral sex on a vagina (your mouth on their vagina)? *This can be with or without orgasm.*

- Daily to several times a day (0)
- Several times a week (1)
- About once a week (2)
- About once a month (3)
- At least once a year (4)
- Prefer not to answer (-9)

| Page Break |  |
| --- | --- |

Display This Question:

If If In the last 12 months, how many different partners have you eaten out or performed oral sex on (y... Text Response Is Greater Than or Equal to 1

|  |  |
| --- | --- |

I22 In the **last 12 months**, how important was eating your partner(s) out (your mouth on their vagina) to you?
 *This can be with or without orgasm.*

- Not important at all (I don’t need it) (0)
- Slightly important (it’s nice for a change) (1)
- Moderately important (I like doing it regularly) (2)
- Important (I really prefer it) (3)
- Very important (it’s central to my sex life) (4)
- Prefer not to answer (-9)

| Page Break |  |
| --- | --- |

Display This Question:

If If In the last 12 months, how many different partners have you eaten out or performed oral sex on (y... Text Response Is Greater Than or Equal to 1

|  |  |
| --- | --- |

I23 In the **last 12 months**, how often did you use a barrier (like a dental dam or receptive condom/female condom seen below) while eating out your partner(s)? *This can be with or without orgasm.*

- Never (or almost never) (0)
- Seldom (less than 25% of occasions) (1)
- Sometimes (26-50% of occasions) (2)
- Often (51-75% of occasions) (3)
- Usually (76-99% of occasions) (4)
- Always (or almost always) (5)
- Prefer not to answer (-9)

| Page Break |  |
| --- | --- |

Display This Question:

If If In the last 12 months, how many different partners have you eaten out or performed oral sex on (y... Text Response Is Greater Than or Equal to 1

|  |  |
| --- | --- |

I24 In the **last 12 months**, how often did you check your mouth for sores or cuts before or after eating out your partner(s)?
 *This can be with or without orgasm.*

- Never (or almost never) (0)
- Seldom (less than 25% of occasions) (1)
- Sometimes (26-50% of occasions) (2)
- Often (51-75% of occasions) (3)
- Usually (76-99% of occasions) (4)
- Always (or almost always) (5)
- Prefer not to answer (-9)

| Page Break |  |
| --- | --- |

Display This Question:

If In the last 12 months, how often did you check your mouth for sores or cuts before or after eatin... = Seldom (less than 25% of occasions)

Or In the last 12 months, how often did you check your mouth for sores or cuts before or after eatin... = Sometimes (26-50% of occasions)

Or In the last 12 months, how often did you check your mouth for sores or cuts before or after eatin... = Often (51-75% of occasions)

Or In the last 12 months, how often did you check your mouth for sores or cuts before or after eatin... = Usually (76-99% of occasions)

Or In the last 12 months, how often did you check your mouth for sores or cuts before or after eatin... = Always (or almost always)

|  |  |  |
| --- | --- | --- |

I24_b Please tell us all the ways you checked your mouth before or after eating out your partner(s).
 *(Check all that apply)*

- Wash my mouth with mouthwash (0)
- Brush my teeth with toothpaste (1)
- Visually check my mouth in a mirror (2)
- Run my tongue around my mouth to feel for bumps and cuts (3)
- Take a photo ("selfie") of my mouth and examine the picture (4)
- Taste for blood (5)
- Not listed (please specify): (-8) ________________________________________________
- ⊗Prefer not to answer (-9)

| Page Break |  |
| --- | --- |

Display This Question:

If If In the last 12 months, how many different partners have you eaten out or performed oral sex on (y... Text Response Is Greater Than or Equal to 1

|  |  |
| --- | --- |

I25 In the **last 12 months**, how often did you worry about possibly getting a sexually transmitted infection (STI) from eating out your partner(s)?
 *This can be with or without orgasm.*

- Never (or almost never) (0)
- Seldom (less than 25% of occasions) (1)
- Sometimes (26-50% of occasions) (2)
- Often (51-75% of occasions) (3)
- Usually (76-99% of occasions) (4)
- Always (or almost always) (5)
- Prefer not to answer (-9)

| Page Break |  |
| --- | --- |

I_INTRO_RECIPIENT Thanks for answering those questions. We're now going to ask about **your experiences** as the **recipient of sexual acts**.

 To start, we have just a few questions about your experiences **receiving blowjobs**. If you do not have a penis you can answer "none."

| Page Break |  |
| --- | --- |

|  |  |
| --- | --- |

I26 **In your lifetime**, how many different partners have **given you blowjobs or performed oral sex on you** (their mouth on your penis)?

- None (0)
- 1-10 partners (1)
- 11-20 partners (2)
- 21-100 partners (3)
- 101-1,000 partners (4)
- 1,001-10,000 partners (5)
- More than 10,000 partners (6)
- Prefer not to answer (-9)

Skip To: I_INTRO_RECIPIENT2 If In your lifetime, how many different partners have given you blowjobs or performed oral sex on yo... = None

| Page Break |  |
| --- | --- |

|  |  |  |  |
| --- | --- | --- | --- |

I27 In the **last 12 months**, how many different partners have **given you blowjobs or performed oral sex on you** (their mouth on your penis)?
 *This can be blowjobs with or without orgasm.*

- Number of partners (1) ________________________________________________
- None, no partners have given me blowjobs in the last 12 months. (0)
- Prefer not to answer (-9)

| Page Break |  |
| --- | --- |

I_INTRO_RECIPIENT2 The following questions will ask about **your experiences being rimmed** by your partner(s).

| Page Break |  |
| --- | --- |

|  |  |
| --- | --- |

I28 **In your lifetime**, how many different partners **have rimmed you** or performed oral sex on your anus (their mouth on your anus)?

- None (0)
- 1-10 partners (1)
- 11-20 partners (2)
- 21-100 partners (3)
- 101-1,000 partners (4)
- 1,001-10,000 partners (5)
- More than 10,000 partners (6)
- Prefer not to answer (-9)

Skip To: I_INTRO_RECIPIENT3 If In your lifetime, how many different partners have rimmed you or performed oral sex on your anus... = None

| Page Break |  |
| --- | --- |

|  |  |  |  |
| --- | --- | --- | --- |

I29 In the **last 12 months** how many different partners have **rimmed you** or performed oral sex on you (their mouth on your anus)?

- Number of partners (1) ________________________________________________
- None, I have not been rimmed in the last 12 months. (0)
- Prefer not to answer (-9)

| Page Break |  |
| --- | --- |

I_INTRO_RECIPIENT3 The following questions are about **your experiences being eaten out** by your partner(s). If you do not have a vagina you can answer "none."

| Page Break |  |
| --- | --- |

|  |  |
| --- | --- |

I30 **In your lifetime**, how many different partners **have eaten you out** or performed oral sex on you (their mouth on your vagina)?
 *This can be with or without orgasm.*

- None (0)
- 1-10 partners (1)
- 11-20 partners (2)
- 21-100 partners (3)
- 101-1,000 partners (4)
- 1,001-10,000 partners (5)
- More than 10,000 partners (6)
- Prefer not to answer (-9)

Skip To: End of Block If In your lifetime, how many different partners have eaten you out or performed oral sex on you (th... = None

| Page Break |  |
| --- | --- |

|  |  |  |  |
| --- | --- | --- | --- |

I31 In the **last 12 months**, how many different partners **have eaten you out** or performed oral sex on you (their mouth on your vagina)?
 *This can be with or without orgasm.*

- Number of partners (1) ________________________________________________
- None, I have not been eaten out in the last 12 months. (0)
- Prefer not to answer (-9)

End of Block: I_Sexual Behaviors

Start of Block: J_STI

J_INTRO **This section will ask about your personal history of sexually transmitted infections (STIs).**

| Page Break |  |
| --- | --- |

|  |  |  |
| --- | --- | --- |

J01 Have you ever had any of the following sexually transmitted infections (STIs)? *(Check all that apply)*

- Syphilis (1)
- Gonorrhea (2)
- Chlamydia (3)
- Human papillomavirus (HPV) (4)
- Herpes Simplex Virus, HSV (genital or anal) (5)
- Hepatitis A (6)
- Hepatitis B (7)
- Hepatitis C (8)
- HIV/AIDS (9)
- Non-gonococcal urethritis (NGU)/non-specific urethritis (NSU) (10)
- Trichomoniasis (trich) (11)
- Not listed (please specify): (-8) ________________________________________________
- ⊗Never had a sexually transmitted infection (STI) (0)
- ⊗Prefer not to answer (-9)

Skip To: End of Block If Have you ever had any of the following sexually transmitted infections (STIs)? (Check all that ap... = Prefer not to answer

| Page Break |  |
| --- | --- |

|  |  |
| --- | --- |

J02 Has a doctor or other healthcare provider ever told you that you have genital or oral warts?

- Yes (1)
- No (0)
- Don't know/Not sure (2)
- Prefer not to answer (-9)

End of Block: J_STI

Start of Block: K_HistoryOPCA

K_INTRO **This section will ask about your personal history of screening or diagnosis with certain cancers or if you know anyone diagnosed with cancer.**


If you need a break you can save and return later to finish the survey.

| Page Break |  |
| --- | --- |

|  |
| --- |

K01 Have you ever had an Ear, Nose, and Throat (ENT) specialist screen or examine your throat and tonsils?

- Yes (1)
- No (0)
- Don't know/Not sure (2)

| Page Break |  |
| --- | --- |

|  |  |
| --- | --- |

K02 Have you ever been **screened or examined** **for oropharyngeal cancer**? 
 This may include feeling your neck for lumps, checking the back of your tongue and throat, or examining your tonsil area, as highlighted blue below.

- Yes (1)
- No (0)
- Don't know/Not sure (2)
- Prefer not to answer (-9)

| Page Break |  |
| --- | --- |

|  |  |
| --- | --- |

K03 Have you ever been **screened or examined** **for oral cancer**? 
A screening for oral cancer may include examining the front of your tongue, cheeks, gums, and the roof of your mouth.

- Yes (1)
- No (0)
- Don't know/Not sure (2)
- Prefer not to answer (-9)

| Page Break |  |
| --- | --- |

|  |  |
| --- | --- |

K04 Have you ever been **diagnosed** with oropharyngeal **or** oral cancer?

- Yes (1)
- No (0)
- Prefer not to answer (-9)

| Page Break |  |
| --- | --- |

Display This Question:

If Have you ever been diagnosed with oropharyngeal or oral cancer? = Yes

|  |  |
| --- | --- |

K04_b In which **year** were you **diagnosed** with oropharyngeal or oral cancer?

▼ 1960 (1) ... Prefer not to answer (-9)

| Page Break |  |
| --- | --- |

Display This Question:

If Have you ever been diagnosed with oropharyngeal or oral cancer? = Yes

K04_c Is there anything you would like the research team to know about your experience(s) with oropharyngeal or oral cancer screenings or diagnoses?

________________________________________________________________

________________________________________________________________

________________________________________________________________

________________________________________________________________

________________________________________________________________

| Page Break |  |
| --- | --- |

|  |  |
| --- | --- |

K05 Have you ever been **diagnosed** with anal cancer or pre-cancer (dysplasia)?

- Yes (1)
- No (0)
- Prefer not to answer (-9)

| Page Break |  |
| --- | --- |

K_INTRO2 **The next questions will ask if you know or have known anyone diagnosed with certain cancers.**

| Page Break |  |
| --- | --- |

|  |  |
| --- | --- |

K06 Have you known anyone **diagnosed** with oropharyngeal cancer? (Such as a friend, partner or family member)

- Yes (1)
- No (0)
- Prefer not to answer (-9)

| Page Break |  |
| --- | --- |

|  |  |
| --- | --- |

K07 Have you known anyone **diagnosed** with oral cancer? (Such as a friend, partner or family member)

- Yes (1)
- No (0)
- Prefer not to answer (-9)

| Page Break |  |
| --- | --- |

|  |  |
| --- | --- |

K08 Have you known anyone **diagnosed** with anal cancer? (Such as a friend, partner or family member)

- Yes (1)
- No (0)
- Prefer not to answer (-9)

End of Block: K_HistoryOPCA

Start of Block: L_Needs Assessment

L_INTRO **Moving on to a new section. These questions will begin asking about things you might need for a hypothetical screening at home.**
 
You are 3/4 of the way through the survey!

| Page Break |  |
| --- | --- |

|  |  |
| --- | --- |

L01 Do you know how you would start a conversation with **a doctor** about checking you for oropharyngeal or oral cancer?

- Yes (1)
- No (0)
- Prefer not to answer (-9)

| Page Break |  |
| --- | --- |

|  |  |
| --- | --- |

L02 Do you know how you would start a conversation with **a dentist** about checking you for oropharyngeal or oral cancer?

- Yes (1)
- No (0)
- Prefer not to answer (-9)

| Page Break |  |
| --- | --- |

|  |  |
| --- | --- |

L03 Do you have a phone with a camera?

- Yes (1)
- No (0)
- Prefer not to answer (-9)

| Page Break |  |
| --- | --- |

|  |  |
| --- | --- |

L04 Do you have someone (like a partner, roommate or friend) that could take a photo of the inside of your mouth if you asked?

- Yes (1)
- No (0)
- Prefer not to answer (-9)

| Page Break |  |
| --- | --- |

|  |  |  |
| --- | --- | --- |

L05 Which of the following would you use to be screened for oropharyngeal cancer?
 *(Check all that apply)*

- A **routine** dental or doctor appointment **in-person** (0)
- A **specialty** dental or doctor appointment **in-person** (1)
- A **routine** dental or doctor appointment **virtually or online** (2)
- A **specialty** dental or doctor appointment **virtually or online** (3)
- A community clinic (4)
- A LGBTQ+ specific health center or clinic (5)
- An emergency dental practice (6)
- An emergency room (7)
- An online screening tool or app where you could take an oral selfie and send it to a healthcare provider for examination (8)
- LGBTQ+ specific events, like Pride (9)
- Not listed (please specify): (-8) ________________________________________________
- ⊗None of these (10)
- ⊗Prefer not to answer (-9)

Display This Question:

If Which of the following would you use to be screened for oropharyngeal cancer?(Check all that apply) = None of these

L05_b Why would you **not** use any of previous suggestions to be screened for oropharyngeal cancer?

________________________________________________________________

________________________________________________________________

________________________________________________________________

________________________________________________________________

________________________________________________________________

| Page Break |  |
| --- | --- |

|  |  |  |
| --- | --- | --- |

L06 I would feel comfortable sending a picture of the inside of my mouth to:
 *(Check all that apply)*

- A confidential healthcare website (0)
- A non-confidential healthcare website (like a message board) (1)
- A healthcare app (2)
- My healthcare system (3)
- Not listed (please specify): (-8) ________________________________________________
- ⊗None of the above (4)
- ⊗Prefer not to answer (-9)

Display This Question:

If I would feel comfortable sending a picture of the inside of my mouth to:(Check all that apply) = None of the above

L06_b Why would you **not** feel comfortable sending a picture of the inside of your mouth to any of the previously suggested options?

________________________________________________________________

________________________________________________________________

________________________________________________________________

________________________________________________________________

________________________________________________________________

End of Block: L_Needs Assessment

Start of Block: M_Hypothetical Screening

M_INTRO **This section is going to continue asking about hypothetical screening and healthcare.**   We appreciate your participation so far! Please keep going!

| Page Break |  |
| --- | --- |

|  |  |
| --- | --- |

M01 I think self-screening for oropharyngeal cancer is something I would use.

- Strongly agree (4)
- Somewhat agree (3)
- Neither agree nor disagree (2)
- Somewhat disagree (1)
- Strongly disagree (0)
- Prefer not to answer (-9)

| Page Break |  |
| --- | --- |

|  |  |
| --- | --- |

M02 I would trust the results of a cancer screening I completed at home by myself **just as much as** a cancer screening completed by a healthcare provider (like a doctor or dentist).

- Strongly agree (4)
- Somewhat agree (3)
- Neither agree nor disagree (2)
- Somewhat disagree (1)
- Strongly disagree (0)
- Prefer not to answer (-9)

| Page Break |  |
| --- | --- |

|  |  |
| --- | --- |

M03 I would feel comfortable having someone **I do not know** teach me how to take a picture of the inside of my mouth in-person. *(With safety measures taken to prevent COVID-19)*

- Strongly agree (4)
- Somewhat agree (3)
- Neither agree nor disagree (2)
- Somewhat disagree (1)
- Strongly disagree (0)
- Prefer not to answer (-9)

| Page Break |  |
| --- | --- |

|  |  |
| --- | --- |

M04 I would feel comfortable having someone **I know** teach me how to take a picture of the inside of my mouth in-person. *(With safety measures taken to prevent COVID-19)*

- Strongly agree (4)
- Somewhat agree (3)
- Neither agree nor disagree (2)
- Somewhat disagree (1)
- Strongly disagree (0)
- Prefer not to answer (-9)

| Page Break |  |
| --- | --- |

|  |  |
| --- | --- |

M05 I would feel comfortable having someone **I know** take a picture of the inside of my mouth.

- Strongly agree (4)
- Somewhat agree (3)
- Neither agree nor disagree (2)
- Somewhat disagree (1)
- Strongly disagree (0)
- Prefer not to answer (-9)

| Page Break |  |
| --- | --- |

|  |  |
| --- | --- |

M06 I am confident I can take a clear picture ("selfie") of the inside of my mouth.

- Strongly agree (4)
- Somewhat agree (3)
- Neither agree nor disagree (2)
- Somewhat disagree (1)
- Strongly disagree (0)
- Prefer not to answer (-9)

| Page Break |  |
| --- | --- |

|  |  |
| --- | --- |

M07 I do not feel the need to be screened for oral or oropharyngeal cancer.

- Strongly agree (4)
- Somewhat agree (3)
- Neither agree nor disagree (2)
- Somewhat disagree (1)
- Strongly disagree (0)
- Prefer not to answer (-9)

| Page Break |  |
| --- | --- |

|  |  |
| --- | --- |

M08 I do not have enough time for oral or oropharyngeal cancer screenings.

- Strongly agree (4)
- Somewhat agree (3)
- Neither agree nor disagree (2)
- Somewhat disagree (1)
- Strongly disagree (0)
- Prefer not to answer (-9)

| Page Break |  |
| --- | --- |

|  |  |
| --- | --- |

M09 There needs to be a website with more information on oropharyngeal cancer in gay and bisexual men.

- Strongly agree (4)
- Somewhat agree (3)
- Neither agree nor disagree (2)
- Somewhat disagree (1)
- Strongly disagree (0)
- Prefer not to answer (-9)

| Page Break |  |
| --- | --- |

|  |  |
| --- | --- |

M10 Free oropharyngeal cancer screening is needed.

- Strongly agree (4)
- Somewhat agree (3)
- Neither agree nor disagree (2)
- Somewhat disagree (1)
- Strongly disagree (0)
- Prefer not to answer (-9)

| Page Break |  |
| --- | --- |

|  |  |
| --- | --- |

M11 I am afraid of dental examinations.

- Strongly agree (4)
- Somewhat agree (3)
- Neither agree nor disagree (2)
- Somewhat disagree (1)
- Strongly disagree (0)
- Prefer not to answer (-9)

| Page Break |  |
| --- | --- |

|  |  |
| --- | --- |

M12 I would **feel comfortable** receiving vaccinations from a dentist.

- Strongly agree (4)
- Somewhat agree (3)
- Neither agree nor disagree (2)
- Somewhat disagree (1)
- Strongly disagree (0)
- Prefer not to answer (-9)

| Page Break |  |
| --- | --- |

|  |  |
| --- | --- |

M13 I would **prefer** to receive vaccinations, like the HPV vaccine, from:

- A dentist or dental practice (4)
- A physician or doctor's office (3)
- A pharmacist or pharmacy (5)
- No preference who I receive vaccinations from (1)
- I prefer to not receive any receive vaccinations (0)
- Prefer not to answer (-9)

| Page Break |  |
| --- | --- |

|  |  |
| --- | --- |

M14 I would prefer to talk about **oral sex and sexual health-related** concerns with a **nurse rather than a doctor**.

- Strongly agree (4)
- Somewhat agree (3)
- Neither agree nor disagree (2)
- Somewhat disagree (1)
- Strongly disagree (0)
- Prefer not to answer (-9)

| Page Break |  |
| --- | --- |

|  |  |
| --- | --- |

M15 I would prefer to talk about **oral sex and sexual health-related** concerns with a **dental hygienist rather than a dentist**.

- Strongly agree (4)
- Somewhat agree (3)
- Neither agree nor disagree (2)
- Somewhat disagree (1)
- Strongly disagree (0)
- Prefer not to answer (-9)

| Page Break |  |
| --- | --- |

|  |  |
| --- | --- |

M16 For my **dental care**, I prefer:

- A male dentist (4)
- A female dentist (3)
- A non-binary or gender non-conforming dentist (1)
- The gender of the dentist does not matter to me (0)
- Prefer not to answer (-9)

| Page Break |  |
| --- | --- |

|  |  |
| --- | --- |

M17 For my **healthcare**, I prefer:

- A male doctor (4)
- A female doctor (3)
- A non-binary or gender non-conforming doctor (1)
- The gender of the doctor does not matter to me (0)
- Prefer not to answer (-9)

| Page Break |  |
| --- | --- |

|  |  |
| --- | --- |

M18 **After taking this survey,** which of the following best describes where you are at right now with regard to checking for oropharyngeal cancer?
 *(Checking for oropharyngeal cancer can mean either checking yourself or having a health professional check you)*

- I am **not** thinking about checking for oropharyngeal cancer regularly (4)
- I am thinking about checking for oropharyngeal cancer regularly (1)
- I am preparing to start checking for oropharyngeal cancer regularly (2)
- I am already checking for oropharyngeal cancer regularly (3)
- Prefer not to answer (-9)

Display This Question:

If After taking this survey, which of the following best describes where you are at right now with r... = I am already checking for oropharyngeal cancer regularly

|  |  |
| --- | --- |

M18_b Do you intend to keep checking for oropharyngeal cancer regularly?

- Yes (1)
- No (0)
- Prefer not to answer (-9)

End of Block: M_Hypothetical Screening

Start of Block: N_Knowledge and Income

N_INTRO **Circling back to a few knowledge questions. We appreciate your honest answers.**

| Page Break |  |
| --- | --- |

|  |  |
| --- | --- |

N01 If caught early, HPV-related oropharyngeal cancer has a very high survival rate.

- True (1)
- False (0)
- Don't know/Not sure (2)
- Prefer not to answer (-9)

| Page Break |  |
| --- | --- |

|  |  |
| --- | --- |

N02 Bad oral health (aching teeth and bleeding gums) can lead to oropharyngeal cancer.

- True (1)
- False (0)
- Don't know/Not sure (2)
- Prefer not to answer (-9)

| Page Break |  |
| --- | --- |

|  |  |
| --- | --- |

N03 Oropharyngeal cancer is more common in women than in men.

- True (1)
- False (0)
- Don't know/Not sure (2)
- Prefer not to answer (-9)

| Page Break |  |
| --- | --- |

|  |  |
| --- | --- |

N04 HPV-16 is the same virus that causes cervical cancer and most anal cancers.

- True (1)
- False (0)
- Don't know/Not sure (2)
- Prefer not to answer (-9)

| Page Break |  |
| --- | --- |

|  |  |
| --- | --- |

N05 Oropharyngeal cancer is typically diagnosed through visual inspection of the mouth and throat.

- True (1)
- False (0)
- Don't know/Not sure (2)
- Prefer not to answer (-9)

| Page Break |  |
| --- | --- |

|  |  |
| --- | --- |

N06 The widely available HPV vaccine series will protect against most types of oropharyngeal cancer if taken in adolescence or early adulthood.

- True (1)
- False (0)
- Don't know/Not sure (2)
- Prefer not to answer (-9)

| Page Break |  |
| --- | --- |

|  |  |
| --- | --- |

N07 Oropharyngeal cancer is usually detected only when it causes symptoms, like problems swallowing or swelling in the neck.

- True (1)
- False (0)
- Don't know/Not sure (2)
- Prefer not to answer (-9)

| Page Break |  |
| --- | --- |

N_INTRO2.5 You are done with knowledge questions!

| Page Break |  |
| --- | --- |

N_INTRO3 **Just a few more questions about yourself. Please keep going!**

| Page Break |  |
| --- | --- |

|  |
| --- |

N08 In what year were you born?
 *(Please enter as a four-digit year, example: 1990)*

________________________________________________________________

| Page Break |  |
| --- | --- |

|  |  |  |  |
| --- | --- | --- | --- |

N09 What is your **approximate** **yearly** take-home income? This includes **only your income**. *(If no income, enter 0)*

- $ (1) ________________________________________________
- Prefer not to answer (-9)

| Page Break |  |
| --- | --- |

|  |  |  |  |
| --- | --- | --- | --- |

N10 What is your **approximate** **total household** **yearly** take-home household income? This includes **you and others in your household**, if applicable. *(If no income, enter 0)*

- $ (1) ________________________________________________
- Prefer not to answer (-9)

End of Block: N_Knowledge and Income

Start of Block: O_Insurance

O_INTRO **The next questions are about your health insurance and dental insurance.**This is the last survey section!

| Page Break |  |
| --- | --- |

|  |  |
| --- | --- |

O01 Do you currently have health insurance?

- Yes (1)
- No (0)
- Don't know/Not sure (2)
- Prefer not to answer (-9)

| Page Break |  |
| --- | --- |

Display This Question:

If Do you currently have health insurance? = Yes

|  |  |  |
| --- | --- | --- |

O01_b How did you obtain your current health insurance?
 *(Check all that apply)*

- Employer (mine or my spouse/partner's) (0)
- Through a health insurance marketplace (1)
- Directly from the insurance company, not through the marketplace (2)
- Medicaid (3)
- Medicare (4)
- Through a government program **other than** Medicaid/Medicare (e.g., VA, Tricare, SSI) (5)
- Through a university (student health insurance) (6)
- Through a parent (7)
- Not listed (please specify): (-8) ________________________________________________
- ⊗Don't know/Not sure (9)
- ⊗Prefer not to answer (-9)

| Page Break |  |
| --- | --- |

|  |  |
| --- | --- |

O02 Do you currently have dental insurance?

- Yes (1)
- No (0)
- Don't know/Not sure (2)
- Prefer not to answer (-9)

| Page Break |  |
| --- | --- |

Display This Question:

If Do you currently have dental insurance? = Yes

|  |  |  |
| --- | --- | --- |

O02_b How did you obtain your current dental insurance?
 *(Check all that apply)*

- Employer (mine or my spouse/partner's) (0)
- Through a health insurance marketplace (1)
- Directly from the insurance company, not through the marketplace (2)
- Medicaid (3)
- Medicare (4)
- Through a government program **other than** Medicaid/Medicare (e.g., VA, Tricare, SSI) (5)
- Through a university (student health insurance) (6)
- Through a parent (7)
- Not listed (please specify): (-7) ________________________________________________
- ⊗Don't know/Not sure (8)
- ⊗Prefer not to answer (-9)

End of Block: O_Insurance

Start of Block: P_Evaluation

|  |  |
| --- | --- |

P01 **Well done! You finished the survey!**   **How was this survey for you? Do you think it was:**

- Much too long (4)
- A bit long (3)
- The right length (2)
- A bit short (1)
- Much too short (0)

| Page Break |  |
| --- | --- |

P02 Is there anything else you would like the research team to know?

________________________________________________________________

________________________________________________________________

________________________________________________________________

________________________________________________________________

________________________________________________________________

End of Block: P_Evaluation

Start of Block: Q_Incentive

|  |  |
| --- | --- |

Q01 As a thank you for participating, we would like to give you a $50.00 Amazon e-gift card that will be sent to you through email. Would you like a gift card?

- Yes (1)
- No (0)

Skip To: End of Block If As a thank you for participating, we would like to give you a $50.00 Amazon e-gift card that will... = No

Skip To: Q02 If As a thank you for participating, we would like to give you a $50.00 Amazon e-gift card that will... = Yes

|  |
| --- |

Q02 Please enter the email address you would like the e-gift card sent to.

________________________________________________________________

Display This Question:

If If Please enter the email address you would like the e-gift card sent to. Text Response Is Not Empty

And As a thank you for participating, we would like to give you a $50.00 Amazon e-gift card that will... = Yes

Q_INTRO2 You can expect to receive your e-gift card to the email address you provided within 5-7 business days from orca@umn.edu. If you do not receive your e-gift card within the next 5-7 business days, please contact orca@umn.edu for assistance. It is recommended that you screenshot this page for your records.

| Page Break |  |
| --- | --- |

|  |  |
| --- | --- |

Q03 Based on your responses, we *may* be interested in contacting you in the future about a second **paid** online study opportunity. You would be asked to follow instructions to take a picture of the inside of your mouth (or have someone else help you). The photo would be evaluated by a healthcare provider.   May we possibly contact you about a future research opportunity for this study?

- Yes (1)
- No (0)

Skip To: End of Block If Based on your responses, we may be interested in contacting you in the future about a second paid... = No

| Page Break |  |
| --- | --- |

Display This Question:

If Based on your responses, we may be interested in contacting you in the future about a second paid... = Yes

|  |  |  |  |
| --- | --- | --- | --- |

Q04 Is this the best email to reach you at for a possible future research opportunity? ${Q02/ChoiceTextEntryValue}

- Yes, ${Q02/ChoiceTextEntryValue} is correct. (1)
- No, my correct email address is: (0) ________________________________________________

End of Block: Q_Incentive

Start of Block: R_Submit

R01 Please click **"Submit"** to finish your survey.

End of Block: R_Submit
